# An Approach to Fisher-Rao Metric for Infinite Dimensional Non-Parametric Information Geometry

**DOI:** 10.3390/e28040374

**Published:** 2026-03-25

**Authors:** Bing Cheng, Howell Tong

**Affiliations:** 1 Academy of Mathematics and Systems Science, Chinese Academy of Sciences, Beijing 100190, China; bc2@amss.ac.cn; 2 Department of Statistics, London School of Economics and Political Science, London WC2A 2AE, UK; 3 Department of Statistics and Data Science, Tsinghua University, Beijing 100084, China; 4 Paula and Gregory Chow Institute for the Studies in Economics, Xiamen University, Xiamen 361005, China

**Keywords:** Cramer–Rao Lower Bound, Fishe-r-Rao metric, G-entropy, Kullback–Leibler divergence, information geometry, manifold hypothesis, non-parametric, orthogonal decomposition, Stein score, tangent space

## Abstract

Non-parametric information geometry has long faced an “intractability barrier”: in the infinite-dimensional setting, the Fisher–Rao metric is a weak Riemannian metric functional that lacks a bounded inverse, rendering classical optimization and estimation techniques computationally inaccessible. This paper resolves this barrier by building the statistical manifold on the Orlicz space $L_{0}^{\Phi }\left(P_{f}\right)$ (the Pistone–Sempi manifold), which provides the necessary exponential integrability for score functions and a rigorous Fréchet differentiability for the Kullback–Leibler divergence. We introduce a novel *Structural Decomposition of the Tangent Space* ($T_{f}M=S⊕S^{⊥}$), where the infinite-dimensional space is split into a finite-dimensional *covariate subspace* (*S*)—representing the observable system—and its orthogonal complement ($S^{⊥}$). Through this decomposition, we derive the Covariate Fisher Information Matrix (cFIM), denoted as $G_{f}$, which acts as the computable “Hilbertian slice” of the otherwise intractable metric functional. Key theoretical contributions include proving the *Trace Theorem* ($H_{G}\left(f\right)=\mathrm{Tr}\left(G_{f}\right)$) to identify *G-entropy* as a fundamental geometric invariant; demonstrating the *Geometric Invariance* of the Covariate Fisher Information Matrix (cFIM) as a covariant (0,2)-tensor under reparameterization; establishing the cFIM as the local Hessian of the KL-divergence; and characterizing the *Efficiency Standard* through a generalized *Cramer–Rao Lower Bound* for semi-parametric inference within the Orlicz manifold. Furthermore, we demonstrate that this framework provides a formal mathematical justification for the *Manifold Hypothesis*, as the structural decomposition naturally identifies the low-dimensional subspace where information is concentrated. By shifting the focus from the intractable global manifold to the tractable covariate geometry, this framework proves that statistical information is not a property of data alone, but an active geometric interaction between the environment (data), the system (covariate subspace), and the mechanism (Fisher–Rao connection).

## 1. Introduction

### 1.1. Breaking the Intractability Barrier in Infinite Dimensional Non-Parametric Information Geometry

Information geometry treats families of probability distributions as geometric spaces known as *statistical manifolds*. Within this framework, the Fisher–Rao metric stands as the canonical measure of statistical distinguishability, successfully defining a Riemannian structure. Current applications, as advocated by S. Amari (2021) [1] and others, are primarily confined to a finite-dimensional parametric geometry focusing on a Riemannian manifold $M_{\theta }$ indexed by the parameters θ as coordinates. However, transitioning from a finite coordinate system to the non-parametric setting, say M={f}, which is typically an infinite-dimensional manifold, defies simple generalizations. It encounters a formidable barrier of intractability, in which the infinite-dimensional Fisher–Rao metric, $g_{f},$ ceases to be a computable matrix.

To overcome the formidable hurdle, we introduce a novel geometric approach, namely *an orthogonal decomposition of the tangent space* $T_{f}M,$ designed to extract a statistically meaningful coordinate system for the otherwise unwieldy infinite-dimensional manifold. By partitioning the tangent space into observed and residual components, we define the structural partition of the space conceptually as

$$T_{f}M=S⊕S^{⊥},$$

where *S* is a subspace, and $S^{⊥}$ its orthogonal complement under the Fisher–Rao metric. This decomposition furnishes a convenient separation of a manageable statistical signal from unobserved complexity, while ensuring that the infinite-dimensional tangent space closure retains a formal Orlicz space structure. Furthermore, the orthogonal decomposition structurally gains the partitioning of the statistical information into two fundamentally distinct and $g_{f}$-orthogonal components. Specifically, *S*, called the *covariate subspace*, is the finite-dimensional and analytical part that captures all the statistical information due to the observable x. And ($S^{⊥}$), called the *Residual Subspace*, remains infinite-dimensional, consisting of pure noise and unobserved factors. This decomposition is significant because the geometry restricted to *S* (defined by $G_{f}$) provides the statistical relevance needed, for example, for semi-parametric efficiency as well as a proof for G-entropy as a measure of observed information.

Briefly, important benefits of the orthogonal decomposition are as follows:

(i) It provides a powerful weapon to solve various problems and applications of infinite dimensional information geometry. (ii) By leveraging the unified framework, we develop a powerful tool based on the matrix $G_{f}$ for inference, targeting the observed manifold. Unlike traditional parametric Fisher information, which is often intractable in high-dimensional settings, $G_{f}$ provides a finite, computable representation of the metric restricted solely to observable covariates. Most significantly, we prove that the G-entropy $H_{G}\left(f\right),$ first proposed by Cheng and Tong (2024) [2], is actually $Tr(G_{f}).$ This is fundamental. It provides the first rigorous information-geometric foundation for G-entropy, giving it a clear statistical meaning as a measure of *total information* derived directly from the underlying geometry. It confirms the basic role of G-entropy in generative statistics, offering a foundational advance for applications in high-dimensional settings, such as generative AI and diffusion models. (iii) We further demonstrate the utility of $G_{f}$ in *semi-parametric efficiency* and hypothesis testing for the *Manifold Hypothesis*. (iv) We discuss approximation of the KL-divergence by the orthogonal decomposition. The detailed proofs of the lemmas and theorems discussed throughout this paper are provided in Appendix A.

### 1.2. The Non-Parametric Information Riemannian Manifold

While early foundations focused on finite-dimensional models, the modern mathematical characterization of statistical manifolds as specific Riemannian structures is extensively treated by Ay et al. (2017) [3], providing the necessary framework for extending these concepts to the infinite-dimensional setting.

**Definition 1** (The Non-parametric Statistical Manifold and Tangent Space)**.** *Let M be the set of all probability density functions f on $R^{n}$ that are strictly positive and satisfy the Pistone–Sempi regularity conditions (Pistone and Sempi, 1995 [4]). The tangent space $T_{f}M$ at a density f∈M is defined as the space of absolute perturbations h(x) which are the velocities of smooth curves $\gamma \left(t\right)=f_{t}$ passing through f (where γ(0)=f).*

*We equip $T_{f}M$ with the structure of the centered Orlicz space $L_{0}^{\Phi }\left(P_{f}\right)$ via the score mapping s=h/f. The topology is induced by the Luxemburg norm:*

$${∥h∥}_{T_{f}M}={∥h/f∥}_{\Phi }=\inf\left\{k>0:E_{P_{f}}\left[\Phi \left(\frac{h}{kf}\right)\right]\leq 1\right\}$$

*where the Young function is defined as $\Phi \left(x\right)=e^{\left|x\right|}-1-\left|x\right|$ to ensure the existence of all exponential moments.*

To ensure mathematical tractability in the uncountably infinite-dimensional setting, we model the non-parametric manifold as an affine statistical bundle over the Orlicz space $L_{0}^{\Phi }\left(P_{f}\right)$, following the rigorous construction of the Pistone–Sempi manifold (Pistone and Sempi, 1995 [4]) and its recent extensions (Pistone, 2022 [5]). This framework replaces the classical finite-dimensional ’sphere’ intuition with a Banach manifold structure, where the exponential integrability of score functions provides the necessary regularity for defining a *weak Riemannian metric* without requiring the space to be locally compact.

In the infinite-dimensional setting, the Fisher–Rao metric is a weak Riemannian metric, a property that leads to complex global geometry often studied in the context of diffeomorphism groups and hydrodynamics (Khesin et al., 2024) [6]. While the non-completeness of this metric poses an ’intractability barrier’ for classical estimation, it provides the necessary geometric flexibility to model infinite-dimensional probability densities as points on a manifold.

In additional, why the Pistone–Sempi Orlicz Space (Pistone and Sempi, 1995 [4]) is required here is because using “smooth functions” or a standard $L^{2}$ Hilbert space is often insufficient for non-parametric manifolds:

*1.*  **Guarantees the Existence of the Fisher Metric and KL-Divergence**
  *The Young function $\Phi \left(x\right)=e^{\left|x\right|}-1-\left|x\right|$ specifically ensures that the random variables have exponential moments. This guarantees that the Kullback–Leibler (KL) divergence is finite and that the Fisher–Rao metric is a well-defined (though weak) bilinear form.*
*2.*  **Rigorous Foundation for Affine Duality (*e* and *m* connections)**
  - *In the Pistone–Sempi framework, the tangent space (representing the m-connection or mixture structure) and its dual (representing the e-connection or exponential structure) are properly paired.*
  - *This allows us to treat the following Orthogonal Decomposition not just as a geometric trick, but as a split between two mathematically rigorous dual topologies.*
*3.*  **Support for the “Intractability Barrier” Argument**
  *By defining $T_{f}M$ as a Banach manifold (Orlicz space) rather than an Orlicz manifold, we want to address the following issues:*
  - *In a Banach manifold, the Fisher metric does not induce a “strong” Riemannian structure. That is, the metric does not have a bounded inverse on the whole space.*
  - *To provide a the mathematical “proof” of why the metric is intractable globally. It justifies why our projection onto the finite-dimensional covariate subspace S is the only way to obtain a computable, invertible Fisher Information matrix ($G_{f}$).*
*4.*  **Differentiability of the Likelihood Functional**
  *To prove the* **Natural Gradient** *and very important* **G-entropy results***, the log-likelihood must be Fréchet differentiable. The Orlicz space $L_{0}^{\Phi }$ is the “natural” home for log-likelihood ratios.*

**Lemma 1** (The Centered Tangent Constraint)**.** *For any $h\in T_{f}M$, the absolute perturbation must satisfy the zero-integral constraint:*

$$\int_{R^{n}}h\left(x\right)dx=0$$

*In the Orlicz framework, this is equivalent to state that the score function s=h/f belongs to the centered subspace $L_{0}^{\Phi }\left(P_{f}\right)$ where $E_{P_{f}}\left[s\right]=0$.*

From this Lemma, we know that the tangent space is

$$T_{f}M=\left\{h\in L^{\Phi }\left(P_{f}\right)|\int hdx=0\right\}.$$

**Definition 2** (The Stein Score Function)**.** *For any tangent vector $h\in T_{f}M$, the associated Stein score function s is defined by the following mapping:*

$$s\left(x\right)=\frac{h(x)}{f(x)}$$

*This function represents the relative change in the density and is the primary object for calculating statistical information.*

**Lemma 2** (The Score Isomorphism in Orlicz Topology)**.** *The mapping $\Phi :T_{f}M\to L_{0}^{\Phi }\left(P_{f}\right)$ defined by s=Φ(h)=h/f is a topological isomorphism (a continuous linear bijection with a continuous inverse).*

Here, we have successfully established that the tangent space is not just an abstract set of functions but a Banach space modeled on the Orlicz space.

**Definition 3** (The Fisher–Rao Metric Functional)**.** *Let $T_{f}M≅L_{0}^{\Phi }\left(P_{f}\right)$ be the tangent space at f. The Fisher–Rao metric $g_{f}$ is a symmetric bilinear functional $g_{f}:T_{f}M\times T_{f}M\to R$ defined by the duality pairing:*

(1) $$g_{f}\left(h_{1},h_{2}\right)=\int_{R^{n}}\frac{h_{1}\left(x\right)h_{2}\left(x\right)}{f(x)}dx.$$

Hence, in the infinite-dimensional setting, $g_{f}$ is a weak Riemannian metric, providing a pairing between the tangent space (mixture structure) and the cotangent space (exponential structure).

**Lemma 3** (Score Covariance Identity and Integrability)**.** *For any $h_{1},h_{2}\in T_{f}M$, the metric $g_{f}\left(h_{1},h_{2}\right)$ is equivalent to the covariance of their corresponding scores $s_{1},s_{2}\in L_{0}^{\Phi }\left(P_{f}\right)$, and this value is guaranteed to be finite.*

(2) $$g_{f}\left(h_{1},h_{2}\right)=E_{P_{f}}\left[s_{1}s_{2}\right]={\text{Cov}}_{P_{f}}\left(s_{1},s_{2}\right).$$

As we shall see later, the summation in the Fisher Information Matrix in the parametric case becomes, in the non-parametric case, an integration over $R^{n}$ and the indices i,j become continuous variables, say x,y.

**Metric Kernel**: The integral $\int g\left(x\right)h_{1}\left(x\right)h_{2}\left(x\right)\;dx$ can be viewed as the integral of a tensor density g(x)δ(x−y) against the tangent vectors.

$$g_{f}\left(h_{1},h_{2}\right)=\int_{R^{n}}\int_{R^{n}}\underset{\text{Metric}\;\text{Kernel}\;g_{f}\left(x,y\right)}{\underset{︸}{\left[\frac{1}{4f(x)}\delta \left(x-y\right)\right]}}h_{1}\left(x\right)h_{2}\left(y\right)\;dx\;dy$$

where δ(x−y) is the Dirac delta function. This is the formal infinite-dimensional tensor, connecting the “*coordinates”* *x* and *y*, and the Metric Kernel $g_{f}\left(x,y\right)=\frac{\delta (x-y)}{4f(x)}$.

**The Role of** 1/f(x): The factor $\frac{1}{f(x)}$ ensures that the metric is invariant under diffeomorphisms (reparameterizations) of the sample space of x, a property proven by the Čencov’s theorem [7].

- Low Probability implies High Information Weight: If f(x) is very small at a point *x*, a small change h(x) at that point implies a large proportional change $s\left(x\right)=\frac{h(x)}{f(x)}$. The metric assigns a large weight ($\frac{1}{f(x)}$) to such a change, making pdfs that differ in low-probability regions seem far apart, thus being highly informative.
- High Probability implies Low Information Weight: If f(x) is large, a change in h(x) has less impact on the distance. The full Fisher–Rao metric formula is thus a specific, invariant way to define the “length” of a smooth path of pdfs.

### 1.3. A Crucial Geometric Decomposition in Non-Parametric Information Geometry

The structural foundation of information geometry lies in the dualistic relationship between affine connections, a concept pioneered in the parametric setting by Amari (1987) [8], who first introduced dual connections on Hilbert bundles to characterize the geometry of statistical models. This dualistic framework was later systematized into a comprehensive theory of α-connections and dually flat manifolds by Amari and Nagaoka (2000) [9]. In our non-parametric extension, we utilize these dual structures to bridge the gap between the infinite-dimensional Orlicz manifold and the finite-dimensional covariate subspace *S*.

Although the Fisher–Rao metric is the canonical tool for measuring statistical distance, its application in non-parametric/high-dimensional settings is practically unworkable due to the following challenges:

1. **The Theoretical Hurdle: Loss of Fine Structure and Indexing:**
  The first critical hurdle is the loss of a coordinate system. In the space of non-parametric functions, the Fisher–Rao metric transforms from a matrix I(θ) to a metric functional
  $$g_{f}\left(h,h\right)=\int_{R^{n}}\frac{h^{2}\left(x\right)}{f(x)}dx$$
  operating on the infinite-dimensional tangent space $T_{f}M$ for each $h\in T_{f}M$. Since every vector *h* represents a valid intrinsic statistical direction (A “direction” in the tangent space ($T_{f}M$) is formally a tangent vector *h*. Intuitively, *h* represents a way the pdf *f* can be perturbed or changed), the full metric $g_{f}$ measures the information contained in all possible intrinsic directions.
  Being an integral over the entire sample space, the metric $g_{f}$ does not distinguish between purely residual effects and contributions along specific, meaningful statistical directions that are externally defined by a finite set of observable covariates {x}. The resulting geometry is not useful for practical applications. The following is a simple illustration:
  Suppose we are standing at a spot in a city. Then, $g_{f}$ measures the total statistical distance (or effort) required to move from our current spot to any new spot, regardless of whether we walk, drive, or fly. It is a measure of the total change, but it does not tell us how that change occurred; hence, it is a crude measure, providing only the “straight-line” distance, but not the path. A more focused measure should reflect a geometry with clearly marked roads in the city, e.g., “Main Street,” “Highway 101,” etc., and information of how we move along a specific road, so as to track our progress, e.g., we moved 5 miles along the Main Street.
2. **The Computational Hurdle: The Inverse Problem and Unworkable Optimization:**
  The problem of “intractability” is eclipsed by the computational hurdle related to the inverse of the metric. In the parametric setting (Amari’s framework), the inverse of FIM, $I{\left(\theta \right)}^{-1}$, is the cornerstone for calculating the so-called Natural Gradient—the statistically optimal direction for gradient-based optimization. Unlike the Standard Gradient Descent (SGD), Natural Gradient Descent (NGD) uses the parametric Fisher information matrix I(θ) as a Riemannian metric to precondition the gradient. This metric measures the “distance” or dissimilarity between two pdfs in a way that is invariant to how the model is parametrized. See Jiang Hu et al., (2024) [10].
  In the non-parametric setting or generative processes from a latent space such as the diffusion models or various decoders, the inverse of the metric functional $g_{f}$ is a highly complex integral operator. This operator is almost always impossible to invert analytically or even approximate tractably in high dimensions. Consequently, while the full natural gradient is the theoretically ideal direction of steepest statistical descent (the full Natural Gradient), it remains computationally inaccessible. The non-parametric geometry is thus paralysed in practice and only beautiful in theory; it cannot provide a usable optimization tool for modern machine learning algorithms, which desperately needs an efficient gradient pre-conditioning framework.
3. **The Applied Hurdle: Conflation of Information and Loss of Relevance**
  Perhaps the most compelling argument for the orthogonal decomposition lies in the need for statistical relevance and explainability. In most applied contexts, we are not interested in the total statistical information captured by $g_{f}$, which conflates signal, noise, model misspecification, and unobserved factors. Instead, we seek information that is attributable to a finite set of observable covariates x, i.e., the “signal” rather than the “noise”.

## 2. Structural Decomposition of the Tangent Space: Resolving the Intractability of the Fisher–Rao Metric

### Orthogonal Decomposition of the Tangent Space and the Pythagorean Theorem

Henceforth, we assume that the pdf *f* is zero-valued at the boundaries of $R^{n}$, which implies f(x)→0 as ∥x∥→∞. This ensures that the product term in an integration by parts vanishes. To overcome the intractability barrier for the infinite-dimensional case, we introduce a geometric structure derived from observable covariates to partition $T_{f}M$.

**Definition 4** (The Covariate Subspace *S*)**.** *Suppose we have covariates $x=\{x_{1},\ldots ,x_{n}\}$. Define the covariate subspace S by*

$$S=\text{span}\left\{s_{i}\left(x\right)|s_{i}\left(x\right)=\frac{\partial \ln f(x)}{\partial x_{i}},\;i=1,\ldots ,n\right\}=\left\{w^{T}\nabla f|w\in R^{n}\right\}\subset T_{f}M.$$

**Definition 5** (Residual Subspace $S^{⊥}$)**.** *The Residual Subspace $S^{⊥}$ is the orthogonal complement of S with respect to the Fisher–Rao metric, $g_{f}$. It comprises all tangent vectors $\varepsilon \in T_{f}M$ that are $g_{f}$-orthogonal to every vector in S:*

$$S^{⊥}=\left\{\varepsilon \in T_{f}M|g_{f}\left(\varepsilon ,h_{S}\right)=0\;\forall h_{S}\in S\right\}.$$

The residual subspace is normally infinite-dimensional consisting of statistical variation related to unobserved factors, pure noise, and intrinsic structure not spanned by x.

**Lemma 4.**  *S is a subspace of $T_{f}M.$*

**Lemma 5.**  *S is a finite-dimensional (and thus closed) subspace of $T_{f}M$. On this subspace, the metric $g_{f}$ is a strong Riemannian metric.*

**Theorem 1** (The Orthogonal Decomposition Theorem)**.** *In the Orlicz tangent space $T_{f}M$, there exists a unique orthogonal decomposition with respect to the Fisher–Rao metric $g_{f}$ such that*

(3) $$T_{f}M=S⊕S^{⊥},$$

*where S is the finite-dimensional covariate subspace, and $S^{⊥}=\left\{h\in T_{f}M:g_{f}\left(h,s\right)=0,\forall s\in S\right\}$.*

This theorem proves that while the full space $T_{f}M$ is an intractable Banach space (where $g_{f}$ lacks a bounded inverse), the covariate subspace *S* is a complemented Banach space structure where the geometry behaves like classical Euclidean space.

By using the Orlicz topology, the “decomposition” is not just an algebraic trick; it is a topological necessity.

Next, we consider analytic representation problem. To any two $h_{1}$ and $h_{2}$ in $T_{f}M$, we have tangent vectors that are decomposed:

$$h_{1}\left(x\right)=w_{1}^{T}\nabla f\left(x\right)+\varepsilon_{1}\left(x\right)=h_{1,S}\left(x\right)+h_{1,⊥}\left(x\right),$$

$$h_{2}\left(x\right)=w_{2}^{T}\nabla f\left(x\right)+\varepsilon_{2}\left(x\right)=h_{2,S}\left(x\right)+h_{2,⊥}\left(x\right),$$

where $h_{a,S}=w_{a}^{T}\nabla f\in S$ (the covariate subspace), and $h_{a,⊥}=\varepsilon_{a}\in S^{⊥}$ (the orthogonal complement) for a=1,2.

**Lemma 6.**  *Orthogonality of Mixed Terms: $g_{f}\left(h_{1,S},h_{2,⊥}\right)=0$ and $g_{f}\left(h_{1,⊥},h_{2,S}\right)=0$.*

**Lemma 7.**  *$g_{f}\left(h_{1,S},h_{2,S}\right)=w_{1}^{T}G_{f}w_{2}$, where $G_{f}$ is the matrix with entries*

$${\left(G_{f}\right)}_{ij}=g_{f}\left(\frac{\partial f}{\partial x_{i}},\frac{\partial f}{\partial x_{j}}\right).$$

**Corollary 1.**  (4) $${\left(G_{f}\right)}_{ij}=E_{X∼f}\left[s_{i}\left(X\right)s_{j}\left(X\right)\right],$$

where $s_{i}\left(X\right)$ and $s_{j}\left(X\right)$ are *i*-th and *j*-th Stein score functions of logf(x).

**Definition 6.**  *In order to avoid terminological conflict with Fisher Information Matrix I(θ) for parametric pdfs, here we call the matrix $G_{f}={\left({\left(G_{f}\right)}_{ij}\right)}_{n\times n}$ a Covariate Fisher Information Matrix (cFIM) for the information geometry manifold M.*

If we restrict the tangent vectors to *S*, the infinite-dimensional geometry takes on the tractable, observed form of classical parametric geometry in the following theorem.

**Theorem 2** (Analytical Explainability of the Covariate Metric)**.** *Let $h\in T_{f}M$ and $h_{S}$ be its unique orthogonal projection in S and $v_{h}$ be the cross-information vector with entries ${\left(v_{h}\right)}_{j}=g_{f}\left(h,\partial_{j}f\right)$. Let $w_{h}$ be the coefficient vector such that $h_{S}=w_{h}^{T}\nabla f$. The following properties hold.*

*1.*  *The vector $w_{h}$ is the unique solution to the following linear equation: $G_{f}w_{h}=v_{h}$; that is, if f satisfies some mild conditions, then $G_{f}$ will admit an inverse matrix, and*
(5) $$w_{h}=G_{f}^{-1}v_{h}.$$
*2.*  *The metric $g_{f}$ on the projected subspace becomes a finite quadratic form:*
(6) $$g_{f}\left(h_{S},h_{S}\right)=v_{h}^{T}G_{f}^{-1}v_{h}.$$

The above Theorem demonstrates that while the original metric functional $g_{f}$ is a “black box” integral, the metric on the subspace *S* behaves exactly like the Fisher Information Matrix in Amari’s parametric setup.

Note that cFIM allows us to *index* statistical changes using concrete weights $w_{h}$, transforming abstract geometric deviations into an interpretable feature-based framework.

Having established that the metric on *S* is analytically solvable as above, we now present the geometric and statistical consequences of this structure.

**Theorem 3** (Pythagorean Theorem of Information)**.** *Due to the $g_{f}$-orthogonality between S and $S^{⊥}$, the total statistical information of any h decomposes additively:*

(7) $$g_{f}\left(h,h\right)=g_{f}\left(h_{S},h_{S}\right)+g_{f}\left(\varepsilon ,\varepsilon \right).$$

In words, in a non-parametric setup, the total statistical information of the perturbation *h* is precisely the sum of the observed information ($g_{f}\left(h_{S},h_{S}\right)$), like a signal, and the residual information ($g_{f}\left(\varepsilon ,\varepsilon \right)$), like a noise. The ratio $R=g_{f}\left(h_{S},h_{S}\right)/g_{f}\left(h,h\right)$ is termed the *Information Capture Ratio*, representing the ratio of signal to signal plus noise.

By contrast, in a parametric setup, conventional information geometry incurs only the observed part so that it is much easier to address the theory and applications.

## 3. Information Geometric Foundation of G-Entropy

This section is devoted to show that the G-entropy introduced by Cheng and Tong (2024) [2] is a scalar invariant that measures the amount of information conveyed by a non-parametric manifold.

### 3.1. The Trace Theorem and G-Entropy

By restricting the infinite-dimensional geometry to the finite covariate subspace *S*, we can characterize the “total information” of the observed system through a matrix trace.

**Definition 7** (G-Entropy $H_{G}\left(f\right)$)**.** *The G-entropy $H_{G}\left(f\right)$ of a smooth pdf f is the expectation of the squared norm of the Stein score function for the observable covariates $\left\{x_{1},x_{2},\ldots ,x_{n}\right\}$:*

(8) $$H_{G}\left(f\right)=E_{X∼f}{[∥\nabla \log f\left(X\right)∥}^{2}]=\sum_{i=1}^{n}E_{X∼f}\left[{\left(\frac{\partial \log f}{\partial x_{i}}\right)}^{2}\right].$$

*This scalar quantity represents the “information volume” or “curvature” concentrated in the directions of the covariates.*

**Theorem 4** (Identity of G-Entropy)**.** *Let $T_{f}M≅L_{0}^{\Phi }\left(P_{f}\right)$ be the infinite-dimensional Orlicz tangent space. The total observable information curvature, termed G-entropy $H_{G}\left(f\right)$, is the trace of the metric functional $g_{f}$ restricted to the d-dimensional covariate subspace S:*

(9) $$H_{G}\left(f\right)=\mathrm{Tr}\left(G_{f}\right)=\sum_{j=1}^{d}g_{f}\left(h_{j}^{*},\;h_{j}^{*}\right)$$

where $\left\{h_{j}^{*}\right\}$ is an orthonormal basis for S with respect to the Fisher–Rao metric.

The Fisher–Rao metric is uniquely distinguished by its invariance under sufficient statistics, a property that forms the core of the categorical approach to information geometry (Ay et al., 2017 [3]). Our derivation of the G-entropy as the trace of the restricted metric $G_{f}$ preserves this fundamental invariance within the covariate subspace *S*.

From this theorem, we demonstrate the following:

- By defining G-entropy as the trace of a finite-rank operator, we measure the “length” of the scores (exponential connection) using the metric (mixture connection) as a **duality pairing**.
- This theorem proves that $H_{G}\left(f\right)$ is not just a property of the data, but a property of the System (S) and the Mechanism ($g_{f}$). If the manifold were “flat” (as classical statisticians assumed), $G_{f}$ would be a constant identity matrix. The fact that $G_{f}$ varies with *f* proves the “active participation” of the statistical manifold in the information-gathering process.

**Theorem 5** (Geometric Invariance of the cFIM under Covariate Reparameterization)**.** *Let M be the non-parametric statistical manifold modeled on the Orlicz space $L_{0}^{\Phi }\left(P_{f}\right)$. At any point f∈M, let $S\subset T_{f}M$ be the n-dimensional covariate subspace spanned by the Stein scores $\left\{s_{1},\ldots ,s_{n}\right\}$ associated with the covariates $x=(x_{1},\ldots ,x_{n})$. Under a diffeomorphism of the covariates x=ψ(y), the Covariate Fisher Information Matrix (cFIM) $G_{f}^{\left(x\right)}$ transforms to $G_{f}^{\left(y\right)}$ as a covariant tensor of rank 2 according to the following formula:*

(10) $$G_{f}^{\left(y\right)}=J^{T}G_{f}^{\left(x\right)}J,$$

*where J is the Jacobian matrix of the transformation with entries $J_{ij}=\frac{\partial x_{i}}{\partial y_{j}}$. This confirms that the matrix $G_{f}$ is coordinate-dependent.*

*Secondly, the G-entropy $H_{G}\left(f\right)=\mathrm{Tr}\left(G_{f}\right)$ remains invariant under any orthogonal transformation of the covariate basis, identifying it as an intrinsic geometric property of the subspace S within the infinite-dimensional tangent space $T_{f}M$. That is,*

(11) $$Tr\left(G_{f}^{\left(y\right)}\right)\;=\;Tr\left(G_{f}^{\left(y\right)}\right).$$

The invariance of the Fisher–Rao metric is fundamentally linked to the action of the diffeomorphism group on the space of densities, where the metric can be viewed as an information-theoretic analogue of the $L^{2}$ metric on the group of volume-preserving transformations (Khesin et al., 2024 [6]). Our proof of the tensor transformation law for $G_{f}$ ensures that the computable covariate information remains consistent with this global diffeomorphism-invariant structure.

### 3.2. Interpretation as Total Observed Statistical Information

Because the trace is the sum of the eigenvalues, $H_{G}\left(f\right)$ rigorously quantifies the total cumulative curvature or the *total observed statistical information* captured by $\{x_{1},x_{2},\ldots ,x_{n}\}.$ Moving beyond heuristics, Theorem 4 provides a solid geometric foundation for $H_{G}\left(f\right)$:

- **Analytical Explainability of Information:** Just as Theorem 2 shows that the metric $g_{f}\left(h_{S},h_{S}\right)$ is solvable via matrix algebra, the G-entropy shows that the statistical information of the subspace *S* is a simple scalar invariant, representing the sum of the statistical information (*lengths*) along all observable feature directions, namely $\left\{x_{1},x_{2},\ldots ,x_{n}\right\}$.
- **Spectral Decomposition:** In terms of the eigenvalues $\lambda_{k}\left(G_{f}\right)$ of $G_{f}$, the G-entropy represents the total variance captured across all principal statistical directions:
  (12) $$H_{G}\left(f\right)=\sum_{k=1}^{n}\lambda_{k}\left(G_{f}\right).$$
  If the *Manifold Hypothesis (MH)* holds, these eigenvalues decay rapidly, and $H_{G}\left(f\right)$ represents the information concentrated on the core low-dimensional manifold. We shall return to the MH later.

### 3.3. Applications to Generative AI and Gradient Regularization

The analytical link between $H_{G}\left(f\right)$ and $G_{f}$ provides a convenient platform for the use of $H_{G}\left(f\right)$ in generative statistics, such as diffusion models and score matching:

- **Metric for Informational Budget:** $H_{G}\left(f\right)$ measures the *cost* (in terms of information distance) of moving a unit distance in the covariate space. In generative modeling, a high G-entropy indicates a steep, high-information density region where generative algorithms must take precise, small steps to remain on the manifold.
- **Geometric Regularization:** The link justifies using $H_{G}\left(f\right)$ as a regularizer. Minimizing it during the training of score-based models is equivalent to constraining the total information of the score functions, which promotes the learning of *statistically smooth* distributions that are easier to sample via tools such as the Langevin dynamics.
- **Relationship to Preconditioning:** While the matrix $G_{f}^{-1}$ is used to “correct” or precondition the update direction referred to in Theorem 2, the trace $H_{G}\left(f\right)$ provides a robust scalar diagnostic of the local sampling difficulty before the full matrix inverse is calculated.

## 4. Between KL-Divergence, G-Entropy, and Metric Curvature

The notion of Analytical Explainability enables us to demonstrate that $H_{G}\left(f\right)$ and $G_{f}$ are inter-related computable realizations of the local curvature of the Kullback–Leibler (KL) divergence.

### 4.1. The Fisher Information Metric as the Local Hessian of KL-Divergence

Recall that, in Information Geometry, a smooth curve $f_{t}$ on the statistical manifold *M* and its associated tangent vector *h* are defined as follows:

**Definition 8** (Smooth Curve ($f_{t}$))**.** *A smooth curve $f_{t}$ (or f(x,t)) is a one-parameter family of pdfs on the manifold M. This curve must satisfy two conditions for t in a neighborhood of 0:*

*1.*  **Normalization:** *$\int_{R^{n}}f_{t}\left(x\right)dx=1$ for all t.*
*2.*  **Smoothness:** *$f_{t}\left(x\right)$ is a $C^{\infty }$ function of the parameter t. The curve starts at the reference pdf f, so that $f_{t=0}=f$.*

**Definition 9** (Associated Tangent Vector (*h*))**.** *Since the curve $f_{t}$ must preserve normalization for all t, the tangent vector h must satisfy the zero-integral constraint:*

$$\int_{R^{n}}h\left(x\right)dx=\int_{R^{n}}{\left.\frac{\partial f_{t}\left(x\right)}{\partial t}\right|}_{t=0}dx={\left.\frac{\partial }{\partial t}\left(\int_{R^{n}}f_{t}\left(x\right)dx\right)\right|}_{t=0}={\left.\frac{\partial }{\partial t}\left(1\right)\right|}_{t=0}=0.$$

*The tangent vector $h\in T_{f}M$ is defined as the instantaneous rate of change of the pdf along the curve at t=0:*

$$h\left(x\right)={\left.\frac{\partial f_{t}\left(x\right)}{\partial t}\right|}_{t=0}.$$

**Lemma 8** (The Zero First Derivative of KL Divergence)**.**
$${\left.\frac{d}{dt}D_{KL}\left(f|\right|f_{t})\right|}_{t=0}=0.$$

**Lemma 9** (Second Derivative of Log-Likelihood)**.**
(13) $${\left.\frac{\partial^{2}}{\partial t^{2}}\log f_{t}\left(x\right)\right|}_{t=0}={\left.\frac{\partial s_{t}\left(x\right)}{\partial t}\right|}_{t=0}-s{\left(x\right)}^{2},$$

*where $s_{t}\left(x\right)=\frac{\partial \log f_{t}\left(x\right)}{\partial t}$, and $s\left(x\right)=\frac{\partial \log f(x)}{\partial x}$ (the Stein score function).*

**Lemma 10** (Zero Mean of the Second Derivative of the Tangent Density)**.**
$$\int_{R^{n}}\left[{\left.\frac{\partial^{2}f_{t}\left(x\right)}{\partial t^{2}}\right|}_{t=0}\right]dx=0.$$

**Theorem 6** (Fisher Information as the Second Derivative of KL-Divergence)**.**
(14) $$g_{f}\left(h,h\right)={\left.\frac{d^{2}}{dt^{2}}D_{KL}\left(f|\right|f_{t})\right|}_{t=0}.$$

### 4.2. Geometric Identity: G-Entropy as the Sum of KL Hessians

Let us restrict ourselves to *S*. In it, we can link the G-entropy directly to the directional curvatures of the KL divergence along observable paths. Specifically, to evaluate the curvature in the direction of $x_{i}$, we define a smooth curve $f_{i,t}$ that describes an infinitesimal statistical perturbation driven only by the Stein score function $\partial \log f/\partial x_{i}$.

**Definition 10** (Covariate Perturbation Curve $f_{i,t}$)**.** *For each covariate $x_{i}$, we define a smooth curve $f_{i,t}$ starting at f ($f_{i,0}=f$), such that the tangent vector $h_{i}$ at t=0 is proportional to the Stein score function $s_{i}\left(x\right)$:*

$$h_{i}\left(x\right)={\left.\frac{\partial f_{i,t}\left(x\right)}{\partial t}\right|}_{t=0}=f\left(x\right)s_{i}\left(x\right).$$

Since the Stein score function $s_{i}\left(x\right)$ is one of the basis vectors of *S*, this curve defines a geodesic path whose length is governed by the *i*-th diagonal element of $G_{f}$.

**Theorem 7** (G-Entropy and the KL Second Derivative)**.** *The G-entropy $H_{G}\left(f\right)$ is equal to the sum of the second derivatives of the KL divergence $D_{KL}\left(f|\right|f_{i,t})$ evaluated along each covariate perturbation curve $f_{i,t}$.*

(15) $$H_{G}\left(f\right)=\sum_{i=1}^{n}{\left.\frac{d^{2}}{dt^{2}}D_{KL}\left(f|\right|f_{i,t})\right|}_{t=0}.$$

This result provides a deep theoretical grounding for G-entropy: (i) It is the *total cumulative curvature of the KL divergence across all observable covariate dimensions*. (ii) It quantifies the Total Observed Statistical Information as the sum of the local *sensitivities* of the log-likelihood.

### 4.3. The Third-Order Geometry and the Origin of Asymmetry of KL Divergence

While the second derivative (the metric) provides a symmetric measure of distance, the global KL divergence is *inherently asymmetric* ($D_{KL}\left(p||q\right)\neq D_{KL}\left(q||p\right)$). This asymmetry is captured by the third derivative of the divergence, known as the Amari–Chentsov Cubic Tensor T(h,h,h).

The Cubic Tensor measures the statistical *skewness* of the score function and acts as the sole geometric obstruction to a manifold being “dually flat”. It quantifies the *non-Riemannian structure* that causes the exponential connection ($\nabla^{\left(1\right)}$) and the mixture connection ($\nabla^{\left(-1\right)}$) to diverge. Specifically, the difference between forward and reverse KL divergences is determined entirely by this third-order tensor.

In classical differential geometry, the *connection* (∇) defines how to differentiate vector fields along a curve, dictating the manifold’s geodesic paths. In Information Geometry, the non-symmetric nature of the KL divergence necessitates a family of dual connections, known as the α-connections.

**Definition 11** (α-Connection ($\nabla^{\left(\alpha \right)}$))**.** *Note that here we are considering non-parametric statistical manifold M, not the conventional finite-dimensional parametric manifold $M_{\theta }.$*

*The $\nabla^{\left(1\right)}$ connection is defined by the following covariant derivative acting on tangent vectors $h_{1}$ in the direction of $h_{2}$:*

(16) $$\nabla_{h_{2}}^{\left(1\right)}h_{1}=h_{2}\cdot \frac{\partial }{\partial h_{2}}\left(\frac{h_{1}}{f}\right)\cdot f.$$

More practically, using the tangent score function $s_{1}=h_{1}/f$,

(17) $$\nabla_{h_{2}}^{\left(1\right)}h_{1}=h_{2}\cdot \frac{\partial }{\partial h_{2}}\left(\frac{h_{1}}{f}\right)\cdot f+h_{1}\cdot \left(\frac{h_{2}}{f}\right).$$

The $\nabla^{\left(1\right)}$ connection is *torsion-free*, and its geodesics are curves where the score function h/f changes linearly.

**Definition 12** (Mixture Connection ($\nabla^{\left(-1\right)}$))**.** *The $\nabla^{\left(-1\right)}$ connection is defined by the following covariant derivative:*

(18) $$\nabla_{h_{2}}^{\left(-1\right)}h_{1}=h_{2}\cdot \frac{\partial }{\partial h_{2}}\left(\frac{h_{1}}{f}\right)\cdot f+h_{1}\cdot \left(\frac{\partial s_{2}}{\partial h_{2}}\right)\cdot f-\frac{1}{2}h_{1}\cdot h_{2}\cdot \frac{1}{f}.$$

*The $\nabla^{\left(-1\right)}$ connection is torsion-free, and its geodesics are curves where the density function f changes linearly (a “mixture” of densities).*

**Definition 13** (Amari-Chentsov Cubic Tensor (*T*))**.** *The Cubic Tensor T(h,h,h) is the third-order derivative of the KL divergence along the curve $f_{t}$:*

(19) $$T\left(h,h,h\right)={\left.\frac{d^{3}}{dt^{3}}D_{KL}\left(f|\right|f_{t})\right|}_{t=0}.$$

**Lemma 11** (Zero Third-Order Constraint Term)**.** *The integral term involving the third derivative of the curve density $f_{t}$ and the log-density f, which arises in the calculation of the third derivative of the reverse KL divergence, is identically zero at t=0:*

$$C_{3}\equiv \int_{R^{n}}{\left.\frac{\partial^{3}f_{t}\left(x\right)}{\partial t^{3}}\right|}_{t=0}\log f\left(x\right)dx=0.$$

**Lemma 12** (Third Derivative of Reverse KL Divergence)**.** *The third derivative of the reverse KL divergence, $D_{KL}(f_{t}\left||f\right)$, evaluated at t=0, is equal to the negative of the Cubic Tensor:*

$${\left.\frac{d^{3}}{dt^{3}}D_{KL}(f_{t}\left||f\right)\right|}_{t=0}=-T\left(h,h,h\right).$$

**Theorem 8** (Asymmetry Identity of KL Divergence)**.** *The third-order difference between the forward and reverse Kullback–Leibler divergences is entirely determined by the Amari–Chentsov Cubic Tensor T(h,h,h):*

(20) $${\left.\frac{d^{3}}{dt^{3}}D_{KL}\left(f|\right|f_{t})\right|}_{t=0}-{\left.\frac{d^{3}}{dt^{3}}D_{KL}(f_{t}\left||f\right)\right|}_{t=0}=2T\left(h,h,h\right).$$

## 5. The Covariate Semi-Parametric Efficiency Framework

This section establishes the equivalence between ($G_{f}$) and the statistical bound provided by the Efficient Fisher Information Matrix ($I_{eff}$), establishing its role as the canonical semi-parametric efficiency metric.

In the parametric framework, the Cramer–Rao Lower Bound (CRLB) dictates the fundamental limit of statistical estimation. However, in a non-parametric framework, any attempt to develop a similar CRLB is doomed to failure, because here the manifold *M* is infinite-dimensional, so that the variance of an estimator can be influenced by infinitely many ‘nuisance’ directions, typically rendering the Fisher Information operator *non-invertible* and resulting in a zero bound. Mathematically, the *noise* from the unobserved, infinite-dimensional residual space $S^{⊥}$ overwhelms the signal. This is why pure non-parametric estimation is often regarded as an “*ill-posed*” problem in classical inference.

This section demonstrates that $G_{f}$ can provide a complete resolution leading to *Semi-Parametric Efficiency*.

### 5.1. Parametric Space Partition and Nuisance Tangent Space

We begin by applying the principle of orthogonal decomposition to the parameter space Θ to define the statistical target of interest.

**Definition 14** (Parameter Space and Partition)**.** *The parameter space* Θ *for the statistical model/manifold M is defined by the set of all pdfs {f} parametrized by the pair (θ,η):*

(21) Θ={(θ(f),η(f))∣f∈M}.

- **Parameter of Interest (**θ**)***: The finite-dimensional vector $\theta =T\left(f\right)\in R^{d}$, where $T:M\to R^{d}$ is a continuous functional mapping the non-parametric pdf f to the parameter value.*
- **Infinite-Dimensional Nuisance (η)***: The function or collection of functions that defines the residual pdf structure, η=H(f)∈H, e.g., $L^{2}$.*

**Definition 15** (Nuisance Tangent Space $T_{⊥,\eta }$)**.** *The Nuisance Tangent Space ($T_{⊥,\eta }$) is the closed linear span (CLSP) in the Orlicz space $L^{2}\left(f\right)$ of all Stein scores corresponding to local perturbations in the nuisance parameter **η**, holding **θ** fixed:*

$$T_{⊥,\eta }=\text{CLSP}\left\{\frac{\partial \log f(X;\theta ,\eta )}{\partial \eta }\right\}.$$

**Example 1** (Partially Linear Model (PLM) Coefficients)**.** *Consider the data X=(Y,x,z), where Y is the response, x are the covariates of interest, and z are nuisance covariates.*

$$\text{Model}:\;Y=\theta^{T}x+g\left(z\right)+\varepsilon .$$

*where **θ** is a vector of linear coefficients and can be defined by the functional that minimizes the expected squared residual after projecting Y onto the space spanned by x and z.*

*A common definition of θ(f) relies on the property that the error must be orthogonal to x (after projecting out z’s influence):*

$$\theta \left(f\right)\;\text{solves}:\;\;E_{f}\left[\left(Y-\theta^{T}x-E\left[Y|z\right]\right)\cdot \tilde{x}\right]=0,$$

*where $\tilde{x}$ is the part of x orthogonal to the space spanned by g(z). Clearly **θ** is a functional of the joint density f.*

Other examples include average treatment effect in causal inference, generalized linear models with a non-parametric link and others.

### 5.2. Semi-Parametric Bridge and Geometric Alignment

In this sub-section, we move from the purely geometric properties of the manifold *M* to the statistical problem of estimating a finite-dimensional parameter θ in the presence of an infinite-dimensional nuisance η. A bridge is constructed by aligning the statistical optimum (the efficient score) with the geometric observable (the covariate score). Here, we only focus on the efficiency of semi-parametric estimation within a geometric framework.

From the geometric point of view, efficiency is about the curvature of the manifold. A “sharper” curvature (higher $G_{f}$) means the density changes more rapidly with respect to the covariates, thereby heightening the precision of an estimate of θ. Also, statistical consistency is about the direction (-are we hitting the target?), while efficiency is about the spread (-how tight is the cluster around the target?). In this subsection, we show that the tightness is determined by the metric tensor $G_{f}$.

**Definition 16** (The Efficient Score and Efficient Fisher Information Matrix $I_{eff}$)**.** *Let $T:M\to R^{d}$ be a continuous functional such that θ=T(f). In a the semi-parametric model, the tangent space $T_{f}M$ is decomposed into a Nuisance Tangent Space $T_{⊥,\eta }$ and its orthogonal complement called the Efficient Tangent Space.*

*1.*  **Efficient Tangent Space:** *The orthogonal complement of the Nuisance Tangent Space $T_{⊥,\eta }$ with respect to the Fisher–Rao metric $g_{f}$ is the Efficient Tangent Space: $T_{⊥,\eta }^{⊥}$.*
*2.*  **Efficient Score Function ($s_{eff}$):** *The Efficient Score Function for **θ** is the unique element of the Efficient Tangent Space that corresponds to the score for **θ**. It is defined as the projection of the full score $s_{\theta }=\partial \log f/\partial \theta$ onto $T_{⊥,\eta }^{⊥}$:*
  (22) $$s_{eff}=s_{\theta }-{\tilde{s}}_{\eta },$$
  *where ${\tilde{s}}_{\eta }$ is the projection of $s_{\theta }$ onto $T_{⊥,\eta }$ since $s_{\theta }=s_{eff}+{\tilde{s}}_{\eta }$.*
*3.*  **Efficient Fisher Information Matrix (In semi-parametric statistics theory, $I_{eff}^{-1}$ represents the “** *Information Bound* **”—the smallest possible asymptotic variance achievable by any regular estimator (Bickel et al. [11])) ($I_{eff}\left(\theta \right)$)** *: This is the covariance matrix of the Efficient Score:*
(23) $$I_{eff}\left(\theta \right)=E_{f}\left[s_{eff}s_{eff}^{T}\right].$$

**Assumption 1** (The Geometric Alignment Postulate)**.** *We assume the statistical estimation problem is geometrically aligned with the covariate structure of the data. Specifically, we assume the projection of the signal onto the subspace orthogonal to the nuisance is exactly captured by the covariate score:*

(24) $$\;s_{eff,i}=s_{x_{i}}=\frac{\partial \log f(x)}{\partial x_{i}},\;\;\forall i\in \left\{1,\ldots ,d\right\}.$$

Assumption 1 essentially states that the way *θ* affects the distribution is “*mirrored*” by the way the covariates $\left\{x_{i}\right\}$ span the pdf. Let us briefly discuss why the assumption is reasonable from both the statistical and the geometric points of view. First, we note that functional analysis allows us to project Stein scores onto nuisance tangent spaces.

1. **The Statistical Justification:**
  Statistically speaking, the assumption is a statement about the ancillarity of the data coordinates. In semi-parametric theory, the Efficient Score ($s_{eff}$) is the “*clean*” part of the signal—the part of the likelihood that cannot be explained away by any variation in the nuisance η.
  - **Core logic:** In most regression-like problems, we assume that the “noise” (nuisance) is independent of the “features” (covariates). If the pdf of the noise does not change when we move with respect to the covariates, then the sensitivity of the likelihood to the covariates is the signal.
  - **Why is it reasonable?** If $s_{eff}=s_{x}$, it means that the way the data x “spreads/diffuses” the pdf is exactly the way the parameter θ “identifies” the pdf. If this were not true, it would imply that the covariates are “contaminated” by the nuisance parameters, making it impossible to estimate θ without first perfectly modeling the infinite-dimensional η. In practice, we only build models where we believe the covariates provide a clear, interpretable path to the parameter.
2. **The Information Geometry Justification**:
  From the perspective of Information Geometry (Fisher–Rao metric), the Covariate Fisher Information $G_{f}$ is the intrinsic curvature of the data manifold.
  - **Core logic**: Information Geometry treats the pdf *f* as a point on a manifold. The distance between two pdfs is measured by how much the log-density changes.
  - **The Geometric Bridge**: The assumption asserts that the Statistical Manifold (the space of parameters θ) and the Data Manifold (the space of observations x) are locally isometric.
  - **Why is it reasonable ?** In physics and geometry, we expect the laws governing a system to be invariant under coordinate transformations. If our statistical model is “natural,” the information we gain by changing the parameter should be the same as the information we see by looking at the variance of the data coordinates. And $s_{x}$ is the “Geometric Score.” Aligning it with $s_{eff}$ is simply saying that the geometry of the data and the geometry of the information are the same thing.
3. **Why is it true “in most cases”?**
  - **Exponential Families**: In any natural exponential family (Gaussian, Poisson, and Binomial), the sufficient statistics are tied directly to the covariates. In these cases, the derivatives with respect to the parameter and the data are mathematically linked with often just a sign change or a constant scale.
  - **The Information-Dominant Regime**: In high-dimensional statistics and Machine Learning, we assume that the covariates x are “rich” enough to capture the entire structure of the signal. In this regime, the nuisance η becomes “white noise” relative to the “structured signal” of x. When signal and noise are separated this way, the projection of the score onto the nuisance-orthogonal space naturally collapses onto the covariate directions.

**Theorem 9** (The Geometric Efficiency Identity)**.** *Under the assumption of Geometric Alignment, the Covariate Fisher Information Matrix $G_{f}$ is identical to the Efficient Fisher Information Matrix $I_{eff}\left(\theta \right)$:*

(25) $$G_{f}=I_{eff}\left(\theta \right).$$

**Example 2** (The Gaussian Case for geometric efficiency identity)**.** *To demonstrate that Assumption 1 is a natural structural property, consider the Gaussian family X∼N(θ,η) where θ (mean) is the parameter of interest and $\eta =\sigma^{2}$ (variance) is the nuisance.*

*1.*  **The Geometry of the Nuisance** *: The nuisance score $s_{\eta }=\frac{{\left(x-\theta \right)}^{2}}{2\eta^{2}}-\frac{1}{2\eta }$ spans the space of “scale-like” perturbations. In the $L_{0}^{\Phi }\left(P_{f}\right)$ Orlicz space, this score is a quadratic function of (x−θ).*
*2.*  **The Statistical Signal** *: The efficient score $s_{eff}$ must be orthogonal to $s_{\eta }$. In the Gaussian case, the parametric score $s_{\theta }=\frac{x-\theta }{\eta }$ (a linear function) is already orthogonal to the quadratic nuisance score $s_{\eta }$ because the third central moment of a Gaussian is zero. Thus, no projection (subtraction) is needed: $s_{eff}=s_{\theta }$.*
*3.*  **The Geometric Observable** *: The covariate score*
$$s_{x}=\frac{\partial \log f}{\partial x}=-\frac{x-\theta }{\eta }.$$
*4.*  **The Alignment Result** *: We observe that $s_{eff}$ and $s_{x}$ span the exact same one-dimensional subspace in $T_{f}M$. Their magnitudes are identical, and their information content is equivalent:*

$$I_{eff}\left(\theta \right)=E\left[s_{eff}^{2}\right]=\frac{1}{\sigma^{2}},\;G_{f}=E\left[s_{x}^{2}\right]=\frac{1}{\sigma^{2}}.$$

*This example shows that for most commonly used distributions in statistics, the alignment between the optimal estimation direction ($s_{eff}$) and the data-sensitivity direction ($s_{x}$) is a built-in geometric property. The assumption simply generalizes this symmetry to the semi-parametric manifold.*

### 5.3. The Geometry of RAL Estimators and Information Projection by the Tangent Space Decomposition

In semi-parametric theory (See Bickel et al. [11] for example), the RAL (Regular Asymptotically Linear) condition is the bridge that connects an estimator’s performance to the Orlicz space geometry of the tangent space. Without this condition, an estimator could behave “erratically” (like the Hodges’ estimator), making the Cramér–Rao bound meaningless. Next we will show how RAL interacts specifically with our geometric manifold.

An estimator $\hat{\theta }$ is *asymptotically linear* if there exists a function ψ(x), called the *influence function*, such that

$$\sqrt{n}\left(\hat{\theta }-\theta \right)=\frac{1}{\sqrt{n}}\sum_{i=1}^{n}\psi \left(x_{i}\right)+o_{p}\left(1\right).$$

In the $L_{0}^{\Phi }\left(P_{f}\right)$ Orlicz space, the influence function ψ must be an element of the Tangent Space $T_{f}M$. An estimator is *regular* if its limiting distribution is locally stable under small perturbations of the true distribution *P*.

Specifically, consider a “local” sequence of distributions $P_{n,h}$ that approaches *P* at a rate of $1/\sqrt{n}$ along a direction *g* in the tangent space (formally, a Hellinger-differentiable path). An estimator is regular if

$$\sqrt{n}\left({\hat{\theta }}_{n}-\theta \left(P_{n,h}\right)\right)\overset{d}{\to }L\;\mathrm{under}\;P_{n,h},$$

where the limiting distribution *L* is the **same** for every local path *h*.

Regularity rules out “superefficient” estimators like Hodges’ estimator, which performs exceptionally well at one specific parameter value but breaks down completely under the slightest perturbation. In the context of our manifold, regularity ensures the estimator respects the Fisher–Rao metric and does not exploit “twists” in the geometry.

**Definition 17.**  *An RAL estimator is an estimator that satisfies the two distinct mathematical properties: asymptotic linearity and regularity.*

**Lemma 13** (Influence Function Projection)**.** *Let $\hat{\theta }$ be a RAL estimator with influence function $\psi \in T_{f}M$. Under Assumption 1, the asymptotic variance of $\hat{\theta }$ is minimized if and only if $\psi \in S_{x}=S$, where the convariate space S is given in Section 2.*

### 5.4. The Covariate Cramér–Rao Lower Bound (CRLB)

Having established that the optimal influence function must reside in $S_{x}$, we now derive the explicit form of the lower bound.

**Lemma 14** (The Canonical Influence Function)**.** Under Assumption 1, the unique influence function $\psi^{*}$ that minimizes variance in $T_{f}M$ is

$$\psi^{*}=G_{f}^{-1}s_{x}.$$

**Theorem 10** (The Covariate CRLB)**.** *Let $\hat{\theta }$ be any* **RAL** *estimator of **θ**. Under Assumption 1, the asymptotic covariance matrix satisfies*

(26) $$\text{AsyCov}\left(\hat{\theta }\right)⪰G_{f}^{-1},$$

*where $G_{f}$ is the Covariate Fisher Information Matrix, and ⪰ denotes the Loewner partial order on the space of symmetric matrices.*

Now, we propose a procedure to estimate this geometric bound directly from data that may serve as an “efficiency standard” benchmark.

**Geometric Efficiency Estimation Procedure:**

Let $X_{1},\ldots ,X_{n}$ denote an independent sample from an unknown pdf *f*.

1. **Non-Parametric Learning:** Let ${\hat{f}}_{n}\left(x\right)$ denote a consistent estimate of *f* via a non-parametric method (e.g., Kernel Density Estimation).
2. **Geometric Metric Extraction**: Compute the empirical covariate scores ${\hat{s}}_{x}=\nabla_{x}\log{\hat{f}}_{n}$ and the empirical Covariate Information Matrix:
  $${\hat{G}}_{n}=\frac{1}{n}\sum_{i=1}^{n}\left[{\hat{s}}_{x}\left(X_{i}\right){\hat{s}}_{x}{\left(X_{i}\right)}^{T}\right].$$
3. **Bound Calculation**: Compute the Estimated Covariate CRLB: $\hat{\mathrm{CRLB}}={\left[{\hat{G}}_{n}\right]}^{-1}$.
4. **Efficiency Testing**: For a candidate estimator $\hat{\theta }$ with estimated variance $\hat{\mathrm{Var}}\left(\hat{\theta }\right)$, calculate the efficiency ratio:
  $$\mathrm{Eff}=\frac{\mathrm{Trace}(\hat{\mathrm{CRLB}})}{\mathrm{Trace}(\hat{\mathrm{Var}}\left(\hat{\theta }\right))}.$$
  If Eff≈1, the estimator extracts all the geometrically available information.

### 5.5. Existence and Invertibility of the Metric Tensor $G_{f}$

The Covariate Fisher Information Matrix is defined as the Gram matrix of the covariate scores:

$$G_{f}=E_{f}\left[s_{x}s_{x}^{T}\right],$$

where $s_{x}=\nabla_{x}\log f\left(X\right),$ the Stein scores in the tangent space $T_{f}M$.

**Theorem 11** (The Linear Independence Condition)**.** *The matrix $G_{f}$ is invertible (positive definite) if and only if the set of covariate score functions $\left\{s_{x_{1}},\ldots ,s_{x_{d}}\right\}$ is linearly independent in the Orlicz space $L^{2}\left(f\right)$.*

Hence, $G_{f}$ is invertible if and only if $S_{x}$ is linearly independent.

## 6. The Manifold Hypothesis and the Infinite Dimensional Statistical Manifold

The goal of a statistical manifold *M* is to capture the geometry of all possible statistical ramifications. (entropy; randomness). However, the price for this universality is opacity. In applications like manifold learning or shape analysis, we need to define efficient operations (like means, principal components, or clustering) on the manifold. The intractability of the full metric $g_{f}$ means these operations cannot be performed directly on *M*, rendering the manifold a theoretical “black-box” for practical data analysis.

The *Manifold Hypothesis* (MH) is a central axiom/assumption of modern representation learning and high-dimensional data analysis. It posits that high-dimensional data, such as images or linguistic vectors, do not fill the ambient space uniformly but instead lie on or near a low-dimensional manifold $M_{l}$. While this hypothesis justifies the use of dimensionality reduction and deep neural networks, it is often treated as an empirical assumption. It is lacking in geometric properties. We aim to address this issue.

### 6.1. Statistical Observability and Alignment of the Core Manifold $M_{l}$ Under the Manifold Hypothesis (MH)

Following the distinction made by Fefferman et al. (2016) [12], we clarify that the Manifold Hypothesis (MH) relates to the effective support of a real-world data distribution, which we link here to the geometric concentration of information within the covariate subspace *S*.

**Definition 18** (Manifold Hypothesis (MH))**.** *Let $x\in R^{n}$ be the observed data distributed according to the smooth pdf f. The Manifold Hypothesis (MH) asserts that there exists a low-dimensional differentiable manifold $D\subset R^{n}$ with intrinsic dimension d≪n such that the data are highly concentrated on or very near D.*

$$\text{MH}\Leftrightarrow \int_{R^{n}\setminus N_{\delta }\left(D\right)}f\left(x\right)dx\approx 0\;\text{for}\;a\;\text{small}\;\text{neighborhood}\;N_{\delta }\left(D\right),$$

*where $N_{\delta }\left(D\right)$ is the δ-neighborhood of D.*

To use the information-geometric tools on the density *f*, we must define the statistical representation of the geometric manifold *D* and establish the link between its intrinsic information and the observable coordinates.

**Definition 19** (Statistical Signal Manifold Tangent Subspace ($M_{l}$))**.** *Let the geometric manifold D be locally parameterized by the smooth embedding x=r(y), where $y\in R^{d}$ are the intrinsic coordinates (d<n). The Statistical Signal Manifold Tangent Subspace ($M_{l}$) is the subspace of the full tangent space $T_{f}M$ spanned by Stein scores with respect to the intrinsic manifold coordinates y:*

$$M_{l}=\text{span}\left\{u_{1},\ldots ,u_{d}\right\}\subset T_{f}M,$$

*where the basis vectors $u_{j}$ are the intrinsic scores:*

$$u_{j}=\frac{\partial \ln f(x)}{\partial y_{j}},\;\mathrm{for}\;j=1,\ldots d.$$

**Definition 20** (Observable Covariate Subspace (*S*))**.** *The observable covariate subspace (S) is the n-dimensional subspace of $T_{f}M$ spanned by the scores with respect to the ambient data coordinates x:*

$$S=\text{span}\left\{s_{1},\ldots ,s_{n}\right\}\subset T_{f}M,$$

*where $s_{i}=\frac{\partial \ln f(x)}{\partial x_{i}}$.*

For the Fisher–Rao metric decomposition to effectively address the MH, we make the following *feasible assumptions* that mathematically link the unknown low-dimensional signal ($M_{l}$) to the observable ambient information (*S*) and confirm the statistical validity of the MH:

**Assumption 2** (Statistical Sufficiency (Subspace Inclusion))**.**
$$M_{l}\subset S.$$

- **Rationale:** The statistical variation along the low-dimensional manifold *D* (represented by $M_{l}$) must be contained within the information captured by the ambient data coordinates $\left\{x_{i}\right\}$.
- **Justification:** This is guaranteed by the chain rule applied to the scores, which confirms the signal is measurable in the x-coordinates. The basis vectors $u_{j}$ are linear combinations of the $s_{i}$:
  $$u_{j}=\sum_{i=1}^{n}s_{i}\left(\frac{\partial x_{i}}{\partial y_{j}}\right).$$

**Assumption 3** (Statistical Separability (The Core MH Premise))**.** *For any statistically relevant $h\in T_{f}M$, the residual component is negligible:*

$$g_{f}\left(\varepsilon ,\varepsilon \right)\approx 0,$$

*where $\varepsilon =h-\Pi_{M_{l}}\left(h\right)$, and $\Pi_{M_{l}}\left(h\right)$ is the orthogonal projection onto $M_{l}$.*

- **Rationale:** The hypothesis is that the full, infinite-dimensional statistical variation of the density ($T_{f}M$) is effectively captured by the low-dimensional manifold’s tangent space ($M_{l}$). The noise or unexplained variation must be negligible.
- **Justification:** This assumption transforms the geometric MH (“data lives near *D*”) into an information-geometric statement (“all information is contained in $M_{l}$”). This is the key link that allows the geometric decomposition to measure the validity of the MH.

**Assumption 4** (Low-Rank Efficiency of $I_{D}$)**.** *The Covariate Fisher Information Matrix (cFIM), $I_{D}$, of the manifold D is full rank d where*

$${\left(I_{D}\right)}_{jk}=g_{f}\left(u_{j},u_{k}\right)=E_{f}\left[u_{j}u_{k}\right].$$

*This ensures that $M_{l}$ has dimension d and that the statistical information along the manifold is non-degenerate.*

- **Rationale:** The *d* intrinsic coordinates y must be non-redundant. The metric on $M_{l}$ must be full rank *d*, confirming the low dimensionality is **efficient** and not merely an arbitrary choice.

The three core assumptions regarding the Statistical Observability and Alignment of the Core Manifold $M_{l}$ under MH have profound implications for translating the geometric MH into a rigorous, testable statistical framework.

- **Implication of Assumption** 2: Statistical Sufficiency ($M_{l}\subset S$)
  Assumption 2 ensures that the low-dimensional signal space $M_{l}$ (defined by intrinsic parameters y) is entirely contained within the observable covariate space *S* (defined by ambient data x). The implication is statistical observability. The true signal of the manifold is, in principle, perfectly detectable through the high-dimensional data gradients. If this were not true, the intrinsic manifold variations would be statistically invisible in the x-coordinate system, making machine learning impossible without explicitly knowing the manifold embedding r(y). Since the Chain Rule dictates this inclusion, the signal is guaranteed to be measurable within the ambient system.
- **Implication of Assumption 3: Statistical Separability** ($g_{f}\left(\varepsilon ,\varepsilon \right)\approx 0$)
  Assumption 3 is the engine that converts the geometric MH into a quantitative statistical statement. The implication is Information Concentration. By asserting that the information of the residual component ε (orthogonal to $M_{l}$) is negligible, the hypothesis formally states that all relevant statistical information of the density *f* is captured by the low-dimensional signal space $M_{l}$. We can exploit this to construct a practical test for the MH.
- **Implication of Assumption 4: Low-Rank Efficiency** ($\mathrm{rank}(I_{D})=d$)
  Assumption 4 implies that the core dimension *d* is non-redundant. Each of the *d* intrinsic dimensions is statistically necessary and independent, maximizing the information captured per dimension. This leads to the operational implication that the intrinsic dimension *d* of the manifold is precisely given by the effective rank of the relevant Fisher Information Matrix (Lemma 1). By combining this with Assumptions 1 and 2, the problem of finding the unknown dimension *d* of the geometric manifold *D* is simplified to calculating the number of significant eigenvalues of the observable metric $G_{f}$, providing a concrete procedure for intrinsic dimension estimation.

### 6.2. The Statistical Decomposition of Information: Measuring Core Manifold Dominance Under the Manifold Hypothesis

While the Manifold Hypothesis is often treated as an empirical assumption, formal mathematical tests for its validity, such as those proposed by Fefferman et al. (2016) [12], have established that data distributions concentrated on low-dimensional structures can be rigorously identified.

In this paper, we use the established framework as follows:

- **Space:** Tangent space $T_{f}M$, an Orlicz space with inner product $g_{f}\left(u,v\right)=E_{f}\left[uv\right]$.
- **Subspaces:**
  − **Signal Manifold** $M_{l}$ (dimension *d*): $M_{l}=\mathrm{span}\left\{u_{1},\ldots ,u_{d}\right\}$, where $u_{j}=\partial \ln f/\partial y_{j}$.
  − **Covariate Space** *S* (dimension *n*): $S=\mathrm{span}\{s_{1},\ldots ,s_{n}\}$, where $s_{i}=\partial \ln f/\partial x_{i}$.
- **Assumptions:**
  − **A1 (Sufficiency)**: $M_{l}\subset S$.
  − **A2 (Separability/Dominance)**: $g_{f}\left(\varepsilon ,\varepsilon \right)\approx 0$ for $\varepsilon =h-\Pi_{M_{l}}\left(h\right)$, for $h\in T_{f}M$.
  − **A3 (Low-Rank Efficiency):** $\mathrm{rank}(I_{D})=d$.

**Theorem 12** (MH Dominance)**.** *This theorem formalizes Assumption 3 into a rigorous geometric decomposition, which is the starting point for quantifying the MH. For any tangent vector $h\in T_{f}M$, the statistical information (squared Fisher–Rao length) decomposes orthogonally with respect to the statistical signal subspace $M_{l}$:*

(27) $$g_{f}\left(h,h\right)=g_{f}\left(\Pi_{M_{l}}\left(h\right),\Pi_{M_{l}}\left(h\right)\right)+g_{f}\left(\varepsilon ,\varepsilon \right),$$

*where $\Pi_{M_{l}}\left(h\right)$ is the orthogonal projection of h onto $M_{l}$, and $\varepsilon =h-\Pi_{M_{l}}\left(h\right)$ is the residual.*

Hence, the MH is strongly supported if $g_{f}\left(\varepsilon ,\varepsilon \right)\approx 0$, meaning the total information is almost entirely captured by the component in the low-dimensional signal space $M_{l}.$

**Lemma 15** (Effective Manifold Dimension)**.** *Under Assumption 4 ($\text{rank}(I_{D})=d$), the dimension of the statistical signal manifold tangent space is exactly d, and the information along any direction $h_{M_{l}}\in M_{l}$ is given by a quadratic form defined by $I_{D}$.*

The following theorem shows why the rejection of the MH (large $g_{f}\left(\varepsilon ,\varepsilon \right)$) is attributed to the complexity or curvature of the true underlying manifold *M*.

**Theorem 13** (Minimal Residual Information)**.** *The squared length of the residual vector $g_{f}\left(\varepsilon ,\varepsilon \right)$ is the minimum squared statistical distance between the full variation h and the statistical signal manifold tangent space $M_{l}$:*

$$g_{f}\left(\varepsilon ,\varepsilon \right)=\min_{v\in M_{l}}g_{f}\left(h-v,h-v\right).$$

*A large $g_{f}\left(\varepsilon ,\varepsilon \right)$ indicates that h cannot be well-approximated by any linear combination of the intrinsic Stein scores $\left\{u_{j}\right\}$, signifying that the density change is highly non-linear relative to the $M_{l}$ structure.*

We now discuss the relationship between the true signal manifold $M_{l}$ and the observable covariate space *S*.

**Lemma 16** (Relationship Between $M_{l}$ and *S* Projections)**.** *Under Assumption 2 ($M_{l}\subset S$), the orthogonal projection of any signal vector $v\in M_{l}$ onto the covariate space S is the vector itself.*

$$\Pi_{S}\left(v\right)=v\;\text{for}\;\text{all}\;v\in M_{l}.$$

**Theorem 14** (Equivalence of Projections under MH Dominance)**.** *Under the combined assumptions of MH Dominance and Subspace Inclusion, the signal captured by the intrinsic manifold ($M_{l}$) is equivalent to the signal captured by the observable covariate space (S). If Assumption 3 holds, then*

$$\Pi_{M_{l}}\left(h\right)\approx \Pi_{S}\left(h\right),$$

*for all $h\in T_{f}M.$*

This Theorem indicates that if the MH is true (Assumption 3), then the low-dimensional signal ($M_{l}$) is the dominant component, making the n−d extra dimensions of the observable space *S* statistically redundant. This simplifies the study of the MH to one of identifying the rank of the metric on $M_{l}$.

#### 6.2.1. Relationship Between the Signal Tangent Space $M_{l}$ and the Covariate Space *S*

The relationship between $M_{l}$ and *S* is one of subspace inclusion and statistical equivalence under the Manifold Hypothesis (MH).

- **Conclusion 1: Subspace Inclusion and Trivial Projection**
  The relationship is primarily defined by Assumption 2 ($M_{l}\subset S$), which is a consequence of the Chain Rule applied to the scores: the statistical signal space $M_{l}$ is a subspace of the observable covariate space *S*.
  As proven by the Lemma 14, the projection of the signal space $M_{l}$ onto the covariate space *S* is trivial (i.e., the signal space itself), confirming that the entire signal is fully contained within the observable coordinates.
- **Conclusion 2: Statistical Equivalence and Redundancy**
  The relationship is further refined by Assumption 3 ($g_{f}\left(\varepsilon ,\varepsilon \right)\approx 0$) and Theorem 12.
  The space *S* can be orthogonally decomposed with respect to $M_{l}$:
  $$S=M_{l}⊕M_{\mathrm{Redundant}},$$
  where $M_{\mathrm{Redundant}}=S\cap M_{l}^{⊥}$ is the (n−d)-dimensional subspace of *S* orthogonal to $M_{l}$.
  **Statistical Implication:** Under the MH (Assumption 3), the statistical information content of the redundant space $M_{\mathrm{Redundant}}$ is negligible. The tangent vector $\Pi_{S}\left(h\right)$ captures all the measurable information, but Theorem 3 shows that the difference between this full observable information $\Pi_{S}\left(h\right)$ and the intrinsic manifold information $\Pi_{M_{l}}\left(h\right)$ is statistically negligible. In essence, $M_{l}$ is the statistically efficient core of *S*.

#### 6.2.2. Relationship Between the Two Covariate Fisher Information Matrices

We have defined two Fisher Information Matrices (FIMs):

1. Intrinsic cFIM on $M_{l}$ (The Metric on *D*):
  $$I_{D}={\left(g_{f}\left(u_{j},u_{k}\right)\right)}_{d\times d},$$
  where $u_{j}=\frac{\partial \ln f(y)}{\partial y_{j}}\in M_{l}.$
2. Covariate FIM on *S* (The Metric on $R^{n}$):
  $$G_{f}={\left(g_{f}\left(s_{i},s_{k}\right)\right)}_{n\times n},\;\mathrm{where}\;s_{i}=\frac{\partial \ln f(x)}{\partial x_{i}}\in S.$$

The relationship between $I_{D}$ and $G_{f}$ is established via the Chain Rule, which forms the basis of Assumption 2.

**Theorem 15** (The Relationship Between $I_{D}$ and $G_{f}$)**.** *The d×d Intrinsic Fisher Information Matrix $I_{D}$ is related to the n×n Covariate Fisher Information Matrix $G_{f}$ by the local embedding Jacobian J=∂x/∂y:*

(28) $$I_{D}=J^{T}G_{f}J.$$

This theorem provides the central result for studying the MH:

Under the MH, the problem of characterizing the Intrinsic FIM ($I_{D}$) (which defines the *d*-dimensional signal manifold) is mathematically equivalent to analyzing the Covariate FIM ($G_{f}$) (which is calculated using only observable x-gradients).

Specifically, by Assumption 3 (Dominance), $G_{f}$ must be rank-deficient with an effective rank *d*. $G_{f}$ is the metric on the ambient space *S*, but its eigenvalues and eigenvectors provide the optimal statistical basis that is spanned by the low-dimensional manifold *D*. The *d* non-zero eigenvalues of $I_{D}$ are exactly related to the *d* large, non-zero eigenvalues of $G_{f}$ through the transformation defined by J.

If the Manifold Hypothesis holds, the eigenvalues of the Covariate FIM ($G_{f}$) decay rapidly, exhibiting a spectral gap that characterizes the intrinsic dimension *d*, consistent with the geometric testing framework of Fefferman et al. (2016) [12].

### 6.3. A New Statistical Methodology for the MH Problem

The new methodology is rooted in Information Geometry (IG).

#### 6.3.1. Testing the Manifold Hypothesis

The core of our test is to check if the statistical information carried by the ambient data gradients (*S*) is effectively restricted to a low-dimensional space ($M_{l}$), as implied by the dominance assumption (Assumption 3).

1. **The Null Hypothesis and Test Statistic**
  The MH is tested as a rank-deficiency hypothesis on the observable Covariate Fisher Information Matrix ($G_{f}$).
  - Null Hypothesis ($H_{0}$): The data pdf *f* is supported by a manifold *D* of intrinsic dimension *d*. Mathematically, this corresponds to the effective rank of the Covariate FIM being *d*:
    $$H_{0}:{\mathrm{rank}}_{\mathrm{eff}}\left(G_{f}\right)=d\ll n.$$
  - Alternative Hypothesis ($H_{1}$): The data pdf *f* is high-dimensional (fully fills $R^{n}$) or the underlying manifold *D* is pathologically curved, meaning the effective rank is high:
    $$H_{1}:{\mathrm{rank}}_{\mathrm{eff}}\left(G_{f}\right)\approx n.$$
2. The **Statistical Procedure (Intrinsic Dimension Estimation)**
  The test procedure mimics Principal Component Analysis (PCA) but uses the Fisher–Rao metric instead of an Euclidean covariance matrix:
  - Estimate the Covariate FIM ($G_{f}$): Since the true pdf *f* is unknown, $G_{f}=E_{f}\left[ss^{T}\right]$ must be estimated from data samples ${\left\{x_{k}\right\}}_{k=1}^{N}$. This requires estimating the Stein score function $s\left(x\right)=\nabla_{x}\ln f\left(x\right)$, often conducted as in Cheng and Tong (2024) [2]. The resulting empirical matrix is ${\hat{G}}_{f}$
  - Perform eigen-decomposition: Decompose the empirical FIM:
    $${\hat{G}}_{f}=\sum_{k=1}^{n}{\hat{\lambda }}_{k}v_{k}v_{k}^{T},$$
    where ${\hat{\lambda }}_{1}\geq {\hat{\lambda }}_{2}\geq \ldots \geq {\hat{\lambda }}_{n}$.
  - Determine the **Intrinsic Dimension** (*d*): The dimension *d* is determined by finding the spectral gap in the eigenvalues. This is the largest gap where ${\hat{\lambda }}_{d}$ is significantly larger than ${\hat{\lambda }}_{d+1}$.
    $$d=\underset{k}{\mathrm{argmax}}\left(\frac{{\hat{\lambda }}_{k}}{{\hat{\lambda }}_{k+1}}\right).$$
    The value *d* is the estimated dimension of the signal space $M_{l}.$
  - Hypothesis Test: The hypothesis is not rejected if d≪n. If the eigenvalues drop off sharply, it supports the MH. A formal statistical test (e.g., a Likelihood Ratio Test variant or a specialized test for rank-deficiency of the FIM) is associated with the probability of observing the remaining information ($\sum_{k=d+1}^{n}{\hat{\lambda }}_{k}$) under the null hypothesis.

#### 6.3.2. Statistical Inference and Manifold Characterization

The eigenvectors and eigenvalues provide statistical results that characterize the manifold *D*.

1. **Statistical Core Coordinates**
  The *d* **dominant eigenvectors** $\left\{v_{1},\ldots ,v_{d}\right\}$ of $G_{f}$ define the basis for the estimated signal space ${\hat{M}}_{l}$.
  - These vectors represent the statistically efficient linear combinations of the Stein scores of the ambient.
  - These are the closest observable representation of the unknown intrinsic Stein scores $u_{j}$ in $M_{l}$ (as implied by Theorem 3, $\Pi_{M_{l}}\left(h\right)\approx \Pi_{S}\left(h\right)$).
  - The magnitudes of the *d* large eigenvalues, ${\hat{\lambda }}_{1},\ldots ,{\hat{\lambda }}_{d}$, quantify the relative importance of each intrinsic dimension, with ${\hat{\lambda }}_{k}$ measuring the statistical length (information content) along the $v_{k}$ direction.
2. **Statistical Quantification of Dominance (Sloppiness)** The theorems quantify how well the MH holds:
  - **Signal Information**: The statistical information contained in the manifold is $E_{\mathrm{Signal}}=\sum_{k=1}^{d}{\hat{\lambda }}_{k}$.
  - **Residual Information (Sloppiness)**: The statistical information residing in the irrelevant/redundant directions (the violation of the MH/Assumption 3) is $E_{\mathrm{Residual}}=\sum_{k=d+1}^{n}{\hat{\lambda }}_{k}$.
  - **Dominance Measure:** The degree of MH dominance is measured by the ratio:
    $$\mathrm{Dominance}\;\mathrm{Ratio}=\frac{E_{\mathrm{Signal}}}{E_{\mathrm{Total}}}=\frac{\sum_{k=1}^{d}{\hat{\lambda }}_{k}}{\sum_{k=1}^{n}{\hat{\lambda }}_{k}}.$$
    A ratio close to 1 strongly supports the MH, indicating that the manifold *D* is statistically sufficient (Assumption 3).
3. **Geometric Interpretation (Curvature and Error)**
  - The residual $E_{\mathrm{Residual}}$ directly relates to the minimum error $g_{f}\left(\varepsilon ,\varepsilon \right)$ that results from approximating a full variation *h* with the *d*-dimensional space $M_{l}$.
  - If $E_{\mathrm{Residual}}$ is small, it implies the underlying distribution is statistically “simple” (low curvature) around the manifold *D*, validating the local linear approximation inherent in the tangent space method. If it is large, the manifold *D* cannot locally describe *f*, suggesting the MH fails in that region.

## 7. Conclusions and Future Research

To overcome the “intractability barrier” inherent in infinite-dimensional non-parametric information geometry, we treat the statistical manifold *M* not as an opaque black box, but as a structure decomposable by observable features, establishing a rigorous framework for *analytical explainability* in high-dimensional statistics.

Our main contribution is the introduction of an *Orthogonal Decomposition of the Tangent Space* ($T_{f}M=S⊕S^{⊥}$). This is a fundamental geometric principle. It allows us to isolate a covariate subspace *S* that transforms the unworkable infinite-dimensional Fisher–Rao metric functional into a finite, computable Covariate Fisher Information Matrix, $G_{f}$. (Due to space limitation, we do not include results of extending the decomposition from the second-order (Fisher–Rao) to the third-order (Cubic Tensor)).

Building on this foundation, we have established five major theoretical advancements as follows.

1. The infinite-dimensional Fisher–Rao metric $g_{f}$ can be orthogonally decomposed, where the metric restricted to the covariate subspace *S* becomes the finite matrix $G_{f}$.
  This resolves the “inverse problem” that paralyzes non-parametric geometry. By providing a computable inverse $G_{f}^{-1}$, we enable the calculation of natural gradients and geometric pre-conditioning in high-dimensional spaces without requiring the full, intractable inverse of the metric functional.
2. The mathematical identity $H_{G}\left(f\right)=\mathrm{Tr}\left(G_{f}\right)$ provides a solid underpinning of the G-entropy of Cheng and Tong (2024) [2] as a basic notion of entropy that is rooted in information geometry. It can be interpreted as a precise regulator of the manifold’s total statistical curvature, validating its use in generative AI (e.g., diffusion models).
3. The G-entropy is equal to the sum of the second derivatives (Hessians) of the KL-divergence along the covariate perturbation curves, thus connecting the local metric structure $G_{f}$ to the global measure (KL-divergence). It implies that minimizing G-entropy is geometrically equivalent to minimizing the sensitivity of the likelihood function to perturbations in the data features, offering a stable objective for robust model training/selection.
4. We establish the Covariate Cramér–Rao Lower Bound, proving that under geometric alignment, $G_{f}$ is *congruent* to the Efficient Fisher Information Matrix ($I_{eff}$). This provides a concrete *Efficiency Standard* for non-parametric estimation. It implies that the matrix $G_{f}$ dictates the fundamental limit of variance for any Regular Asymptotically Linear (RAL) estimator, allowing researchers to benchmark “black-box” estimators against a theoretically derived geometric limit.
5. We check the Manifold Hypothesis with a *statistical test* of rank deficiency. This transforms the Manifold Hypothesis from some vague intuition into a quantifiable property. It implies that the intrinsic dimension of high-dimensional data can be rigorously estimated by the effective rank of $G_{f}$, providing a diagnostic tool to validate or reject the assumption of low-dimensional structure, that is critical to the modern *representation learning*.

### 7.1. Future Direction 1: The Decomposition of Skewness

While the second-order geometry (the metric $g_{f}$) is fully resolved by the orthogonal decomposition, the statistical manifold *M* is generally curved, meaning its higher-order geometry is non-trivial. Our work on the Amari–Chentsov Cubic Tensor *T* (not included here) confirms that this third-order tensor governs the asymmetry (statistical skewness) of the KL divergence.

The natural and essential next step is to extend the decomposition to this third-order object:

$$T=T_{S}+T_{S^{⊥}}+T_{\mathrm{mixed}}.$$

This requires defining a *Covariate Cubic Tensor* ($T_{G}$) that captures the statistical skewness only within the observed subspace *S*.

- The **Covariate Skewness** ($T_{G}$): This third-order tensor would quantify how non-Gaussian (asymmetric) the statistical variations are along the observed covariate directions.
- **Residual Skewness**: This would measure the asymmetry residing entirely in the residual, unobserved space $S^{⊥}$.
- **Mixed Tensors**: These would describe the cross-dependencies between the observed and residual skewness components.

### 7.2. Future Direction 2: Towards a Complete Covariate Dual Geometry

The ultimate goal is to define the full dual geometric structure ($\left\{\nabla^{\left(1\right)},\nabla^{\left(-1\right)},g_{f},T\right\}$) restricted to the covariate subspace *S*. If $T_{G}$ can be calculated, it would allow us to define the Covariate Dual Connections ($\nabla_{G}^{\left(1\right)}$ and $\nabla_{G}^{\left(-1\right)}$) on *S*. This new geometry would have several profound implications:

- **Dual Explainability**: It would provide two distinct, geometrically meaningful ways to measure distance on the manifold (e-geodesics and m-geodesics), both restricted purely to the observable covariate space *S*.
- **Optimal Asymmetric Paths**: It would allow for the optimization of learning algorithms based not just on minimal statistical variance (CRLB) but also on minimal statistical skewness. For instance, it could identify the optimal path $f_{t}$ in parameter space that best maintains the initial distribution’s symmetry properties.
- **Higher-Order Decomposition**: Success in defining $T_{G}$ opens the door to decomposing all higher-order tensors, potentially leading to a complete, infinite-order geometric description of explainability.

In conclusion, the concept of orthogonal decomposition provides the necessary geometric foundation for analytical explainability so that we can use calculus and algebraic methods to study the infinite dimensional information geometry. By successfully defining the second-order structure, we have created a *roadmap* for future work in building a complete, high-order, Covariate Dual Geometry, capable of fully characterizing the complexity, efficiency, and asymmetry of statistical inference in high-dimensional non-parametric systems.

## Data Availability

No new data were created or analyzed in this study. Data sharing is not applicable to this article.

## Appendix A. Proofs of Lemmas and Theorems

**Proof of Lemma 1.** 

1. Let $\gamma \left(t\right)=f_{t}$ be a smooth curve in the manifold *M* such that $f_{0}=f$.
2. By the definition of a probability density, the total mass is preserved for all *t*: $\int_{R^{n}}f_{t}\left(x\right)dx=1$.
3. Differentiating both sides with respect to *t* at t=0:
  $$\frac{\partial }{\partial t}\int_{R^{n}}f_{t}\left(x\right)dx=\int_{R^{n}}\frac{\partial f_{t}\left(x\right)}{\partial t}dx=0.$$
4. By Definition 1, the tangent vector is $h\left(x\right)={\left.\frac{\partial f_{t}}{\partial t}\right|}_{t=0}$. Thus, ∫h(x)dx=0.
5. Substituting h=sf, we have $\int s\left(x\right)f\left(x\right)dx=E_{P_{f}}\left[s\right]=0$. This confirms *h* is centered.

□

**Proof of Lemma 2.** 

1. Linearity: For $h_{1},h_{2}\in T_{f}M$ and a,b∈R,
  $$\Phi \left(ah_{1}+bh_{2}\right)=\frac{ah_{1}+bh_{2}}{f}=a\frac{h_{1}}{f}+b\frac{h_{2}}{f}=a\Phi \left(h_{1}\right)+b\Phi \left(h_{2}\right).$$
2. Bijectivity: * Injectivity: If Φ(h)=0, then h/f=0. Since f>0 almost everywhere, h=0.
3. Surjectivity: For any centered $s\in L_{0}^{\Phi }\left(P_{f}\right)$, let h=sf. By Lemma 1, $\int hdx=E_{P_{f}}\left[s\right]=0$, so *h* is a valid tangent vector in $T_{f}M$.
4. Topological Continuity: By Definition 1, the norm on $T_{f}M$ is defined exactly as the Luxemburg norm of the score ${∥h∥}_{T_{f}M}:={∥h/f∥}_{\Phi }$.
5. Since ${∥h∥}_{T_{f}M}={∥\Phi \left(h\right)∥}_{\Phi }$, the mapping is an isometry. An isometry between Banach spaces is always a topological isomorphism (continuous with a continuous inverse).

□

**Proof of Lemma 3.** 

1. Identity:
  Substituting $h_{1}=s_{1}f$ and $h_{2}=s_{2}f$ into Definition 3:
  $$g_{f}\left(h_{1},h_{2}\right)=\int \frac{\left(s_{1}f\right)\left(s_{2}f\right)}{f}dx=\int s_{1}s_{2}fdx=E_{P_{f}}\left[s_{1}s_{2}\right].$$
2. Mean-Zero:
  By Lemma 1, $E_{P_{f}}\left[s_{1}\right]=E_{P_{f}}\left[s_{2}\right]=0$; thus, $E_{P_{f}}\left[s_{1}s_{2}\right]={\mathrm{Cov}}_{P_{f}}\left(s_{1},s_{2}\right)$.
3. Finiteness (Orlicz Property):
  Since $s_{1},s_{2}\in L_{0}^{\Phi }\left(P_{f}\right)$, they have finite exponential moments. According to the Generalized Hölder’s Inequality for Orlicz spaces, the product $s_{1}s_{2}$ is integrable if $s_{1}\in L^{\Phi }$ and $s_{2}\in L^{\Psi }$ (where Ψ is the conjugate). In the Pistone–Sempi setting, $L^{\Phi }$ is small enough that $s_{1}s_{2}\in L^{1}\left(P_{f}\right)$. This ensures the metric functional is well defined and finite for all vectors in $T_{f}M$.

□

**Proof of Lemma 4.** This requires us to show that $\int_{R^{n}}h_{S}\left(x\right)dx=0$ for all $h_{S}\in S$. Since $h_{S}$ is a linear combination of the basis vectors, it suffices to show that each basis vector is in $T_{f}M$.

$$\int_{R^{n}}\frac{\partial f}{\partial x_{i}}dx=\int_{R^{n-1}}\left(\int_{R}\frac{\partial f}{\partial x_{i}}dx_{i}\right)dx_{\neq i}.$$

By the Fundamental Theorem of Calculus and the assumption that *f* is zero-valued at the boundaries (i.e., f(x)→0 as ∥x∥→∞):

$$\int_{R}\frac{\partial f}{\partial x_{i}}dx_{i}=f\left(x\right){|}_{x_{i}=-\infty }^{x_{i}=+\infty }=0-0=0.$$

Thus, $\int_{R^{n}}\frac{\partial f}{\partial x_{i}}dx=0$. Since *S* is a linear span of such vectors, $S\subset T_{f}M$. □

**Proof of Lemma 5.** The dimension of *S* is at most *n* (if the gradients are linearly independent, dim(S)=n). Any finite-dimensional subspace of a topological vector space (including an Orlicz space) is closed. Since dim(S)≤n<∞, *S* is a closed subspace of $T_{f}M$. □

**Proof of Theorem 1.** 

1. Closedness: *S* is a finite-dimensional subspace of the Banach space $T_{f}M$. In any Hausdorff topological vector space, every finite-dimensional subspace is closed.
2. Existence of Complement (The Banach Case): An arbitrary closed subspace of a Banach space may not be complemented, but every finite-dimensional subspace of a Banach space is complemented.
3. Orthogonality: We define the projection operator $\Pi_{S}:T_{f}M\to S$. For any $h\in T_{f}M$, let $h_{S}=\sum_{j,k}{\left(G_{f}^{-1}\right)}_{jk}g_{f}\left(h,h_{k}\right)h_{j}$. Since $G_{f}$ is a finite-dimensional symmetric positive-definite matrix (and thus boundedly invertible), $h_{S}$ is well defined.
4. Verification: Define $h_{S^{⊥}}=h-h_{S}$. By construction, for any $h_{m}\in S$:
  $$g_{f}\left(h-h_{S},h_{m}\right)=g_{f}\left(h,h_{m}\right)-\sum_{j,k}{\left(G_{f}^{-1}\right)}_{jk}g_{f}\left(h,h_{k}\right)g_{f}\left(h_{j},h_{m}\right)$$
  $$=g_{f}\left(h,h_{m}\right)-\sum_{j,k}{\left(G_{f}^{-1}\right)}_{jk}g_{f}\left(h,h_{k}\right){\left(G_{f}\right)}_{jm}=g_{f}\left(h,h_{m}\right)-g_{f}\left(h,h_{m}\right)=0.$$
5. Uniqueness: The uniqueness follows from the non-degeneracy of the Fisher metric on the finite-dimensional subspace *S*. Thus, $T_{f}M=S⊕S^{⊥}$.

□

**Proof of Lemma 6.** By definition of the orthogonal complement $S^{⊥}$, $h_{2,⊥}=\varepsilon_{2}$ is orthogonal to every vector in *S*, including the basis vectors $\frac{\partial f}{\partial x_{i}}$. Since $h_{1,S}$ is a linear combination of these basis vectors, $h_{1,S}$ must be orthogonal to $h_{2,⊥}$.

$$g_{f}\left(h_{1,S},h_{2,⊥}\right)=g_{f}\left(\sum_{i=1}^{n}w_{1,i}\frac{\partial f}{\partial x_{i}},\varepsilon_{2}\right).$$

By the bi-linearity of the metric $g_{f}$:

$$g_{f}\left(h_{1,S},h_{2,⊥}\right)=\sum_{i=1}^{n}w_{1,i}\cdot g_{f}\left(\frac{\partial f}{\partial x_{i}},\varepsilon_{2}\right).$$

Since $\frac{\partial f}{\partial x_{i}}\in S$ and $\varepsilon_{2}\in S^{⊥}$, the term $g_{f}\left(\frac{\partial f}{\partial x_{i}},\varepsilon_{2}\right)=0$ for all *i*.

$$\Rightarrow g_{f}\left(h_{1,S},h_{2,⊥}\right)=\sum_{i=1}^{n}w_{1,i}\cdot 0=0.$$

The same logic applies to $g_{f}\left(h_{1,⊥},h_{2,S}\right)$, since $h_{1,⊥}=\varepsilon_{1}\in S^{⊥}$ and $h_{2,S}\in S$. □

**Proof of Lemma 7.** Substitute the linear combination form for $h_{1,S}$ and $h_{2,S}$:

$$h_{1,S}\left(x\right)=\sum_{i=1}^{n}w_{1,i}\frac{\partial f}{\partial x_{i}}\;\mathrm{and}\;h_{2,S}\left(x\right)=\sum_{j=1}^{n}w_{2,j}\frac{\partial f}{\partial x_{j}}.$$

Apply the bilinearity of $g_{f}$:

$$g_{f}\left(h_{1,S},h_{2,S}\right)=g_{f}\left(\sum_{i=1}^{n}w_{1,i}\frac{\partial f}{\partial x_{i}},\sum_{j=1}^{n}w_{2,j}\frac{\partial f}{\partial x_{j}}\right),$$

$$g_{f}\left(h_{1,S},h_{2,S}\right)=\sum_{i=1}^{n}\sum_{j=1}^{n}w_{1,i}w_{2,j}\cdot g_{f}\left(\frac{\partial f}{\partial x_{i}},\frac{\partial f}{\partial x_{j}}\right).$$

The term $g_{f}\left(\frac{\partial f}{\partial x_{i}},\frac{\partial f}{\partial x_{j}}\right)$ is, by definition, the (i,j)-th entry of the matrix $G_{f}$. This sum is exactly the definition of a quadratic form in matrix-vector notation:

$$g_{f}\left(h_{1,S},h_{2,S}\right)=w_{1}^{T}G_{f}w_{2}.$$

□

**Proof of Corollary 1.** 

$${\left(G_{f}\right)}_{ij}=g_{f}\left(\frac{\partial f}{\partial x_{i}},\frac{\partial f}{\partial x_{j}}\right)=\int_{R^{n}}\frac{\frac{\partial f}{\partial x_{i}}\frac{\partial f}{\partial x_{j}}}{f(x)}dx$$

$$=\int_{R^{n}}\frac{\partial \log f}{\partial x_{i}}\frac{\partial \log f}{\partial x_{j}}f\left(x\right)dx=E_{X∼f}\left[s_{i}\left(X\right)s_{j}\left(X\right)\right].$$

□

**Proof of Theorem 2.** Start with the definition of $g_{f}\left(h_{S},h_{S}\right)$ and substitute $h_{S}=\sum_{i=1}^{n}w_{i}\left(\partial_{i}f\right)$:

$$g_{f}\left(h_{S},h_{S}\right)=g_{f}\left(h_{S},\sum_{j=1}^{n}w_{j}\left(\partial_{j}f\right)\right).$$

Using linearity and the definition of $v_{h}$:

$$g_{f}\left(h_{S},h_{S}\right)=\sum_{j=1}^{n}w_{j}g_{f}\left(h_{S},\partial_{j}f\right).$$

By the orthogonality condition $g_{f}\left(h-h_{S},\partial_{j}f\right)=0$, we have $g_{f}\left(h_{S},\partial_{j}f\right)=g_{f}\left(h,\partial_{j}f\right)={\left(v_{h}\right)}_{j}$. Substituting this back,

$$g_{f}\left(h_{S},h_{S}\right)=\sum_{j=1}^{n}w_{j}{\left(v_{h}\right)}_{j}=w_{h}^{T}v_{h}.$$

Finally, substitute the solution for $w_{h}$ from Lemma 7 ($w_{h}=G_{f}^{-1}v_{h}$):

$$g_{f}\left(h_{S},h_{S}\right)={\left(G_{f}^{-1}v_{h}\right)}^{T}v_{h}=v_{h}^{T}{\left(G_{f}^{-1}\right)}^{T}v_{h}.$$

Since $G_{f}$ is symmetric, $G_{f}^{-1}$ is also symmetric, so ${\left(G_{f}^{-1}\right)}^{T}=G_{f}^{-1}$:

$$g_{f}\left(h_{S},h_{S}\right)=v_{h}^{T}G_{f}^{-1}v_{h}.$$

The Information Capture Ratio *R* is simply the ratio of the projected information to the total information, as stated. □

**Proof of Theorem 3.** 

1. By the **Existence and Uniqueness of Decomposition Theorem**, any tangent vector *h* can be uniquely written as the sum of its projection onto the covariate subspace *S* and the residual vector:
  $$h=h_{S}+\varepsilon .$$
2. **Expand the Metric:** We calculate the Fisher–Rao metric of *h* with itself, $g_{f}\left(h,h\right)$, using the bilinearity of the metric $g_{f}\left(\cdot ,\cdot \right)$:
  $$g_{f}\left(h,h\right)=g_{f}\left(h_{S}+\varepsilon ,h_{S}+\varepsilon \right).$$
  Expanding this bilinear form:
  $$g_{f}\left(h,h\right)=g_{f}\left(h_{S},h_{S}\right)+g_{f}\left(h_{S},\varepsilon \right)+g_{f}\left(\varepsilon ,h_{S}\right)+g_{f}\left(\varepsilon ,\varepsilon \right).$$
3. **Apply the Orthogonality Condition:** The fundamental definition of the **Covariate Orthogonal Decomposition** is that the two subspaces, *S* and $S^{⊥}$, are $g_{f}$-**orthogonal**. This means the inner product between any vector in *S* and any vector in $S^{⊥}$ is zero. Since $h_{S}\in S$ and $\varepsilon \in S^{⊥}$, we have the orthogonality conditions:
  $$g_{f}\left(h_{S},\varepsilon \right)=0,$$
  $$g_{f}\left(\varepsilon ,h_{S}\right)=0.$$
4. **Simplify the Expanded Metric:** Substituting the orthogonality conditions back into the expanded metric expression from Step 2:
  $$g_{f}\left(h,h\right)=g_{f}\left(h_{S},h_{S}\right)+0+0+g_{f}\left(\varepsilon ,\varepsilon \right).$$
  This simplifies directly to the final additive relationship:
  $$g_{f}\left(h,h\right)=g_{f}\left(h_{S},h_{S}\right)+g_{f}\left(\varepsilon ,\varepsilon \right).$$

□

**Proof of Theorem 4.** 

1. Operator Representation: In the infinite-dimensional Orlicz space, the Fisher–Rao metric $g_{f}$ defines a bounded bilinear form. While the full metric operator on $T_{f}M$ is not trace-class (its trace would be infinite), its restriction to the finite-dimensional subspace *S* (the cFIM $G_{f}$) defines a finite-rank operator $A_{S}:S\to S$.
2. Well-definedness of Trace: A fundamental result in operator theory states that the trace of any operator on a finite-dimensional subspace is well defined and equal to the sum of its eigenvalues.
3. Basis Independence: Let $\left\{s_{j}\right\}$ be the covariate scores. The cFIM $G_{f}$ has entries ${\left(G_{f}\right)}_{jk}=E_{P_{f}}\left[s_{j}s_{k}\right]$. The trace $\mathrm{Tr}\left(G_{f}\right)=\sum_{j=1}^{d}E_{P_{f}}\left[s_{j}^{2}\right]$ represents the sum of the variances of the scores in the chosen coordinate system.
4. Spectral Invariant: Since $S\subset T_{f}M$ and $G_{f}$ is symmetric positive definite, we can find a transformation to an orthonormal basis where $G_{f}$ is diagonal. The trace is invariant under this change of basis within *S*. Thus, $H_{G}\left(f\right)$ is a spectral invariant of the “observable” part of the manifold.

□

**Proof of Theorem 5.** 

1. **Transformation of the Score Basis (Covector Property):**
  In the Orlicz tangent space $T_{f}M$, the basis for the covariate subspace *S* consists of the Stein scores $s_{i}\left(x\right)=\frac{\partial \ln f(x)}{\partial x_{i}}$. Given the coordinate transformation x=ψ(y), we apply the multivariate chain rule to the log-density:
  (A1) $$\tilde{s}j\left(y\right)=\frac{\partial \ln f(\psi (y))}{\partial y_{j}}=\sum_{i=1}^{n}\frac{\partial \ln f(x)}{\partial x_{i}}\frac{\partial x_{i}}{\partial y_{j}}=\sum_{i=1}^{n}s_{i}\left(x\right)J_{ij},$$
  where $J_{ij}$ represents the sensitivity of the *i*-th original coordinate to the *j*-th new coordinate. This proves that the scores transform as the components of a covector field.
2. **Linearity of the Metric Functional:**
  The entries of the cFIM in the *y*-coordinates, ${\left(G_{f}^{\left(y\right)}\right)}_{jk}$, are defined by the Fisher–Rao metric functional $g_{f}$ restricted to the transformed basis $\left\{{\tilde{s}}_{j}\right\}$. By Lemma 3, this is equivalent to the covariance:
  (A2) $$\left(G_{f}^{\left(y\right)}\right)jk=EP_{f}\left[\tilde{s}j\left(y\right)\tilde{s}k\left(y\right)\right].$$
  Substituting the chain rule expansion,
  (A3) $$\left(G_{f}^{\left(y\right)}\right)jk=EP_{f}\left[\left(\sum_{i=1}^{n}s_{i}\left(x\right)J_{ij}\right)\left(\sum_{l=1}^{n}s_{l}\left(x\right)J_{lk}\right)\right].$$
3. **Orlicz Space Integrability and Linearity:**
  Because $s_{i},s_{l}\in L_{0}^{\Phi }\left(P_{f}\right)$, their product is integrable by the generalized Hölder inequality for Orlicz spaces. Since the Jacobian *J* is deterministic with respect to the probability measure $P_{f}$, we pull the summation and the Jacobian terms outside the expectation:
  (A4) $$\left(G_{f}^{\left(y\right)}\right)jk=\sum {i=1}^{n}\sum_{l=1}^{n}J_{ij}E_{P_{f}}\left[s_{i}\left(x\right)s_{l}\left(x\right)\right]J_{lk}.$$
  Recognizing $E_{P_{f}}\left[s_{i}\left(x\right)s_{l}\left(x\right)\right]$ as the elements of the original cFIM matrix ${\left(G_{f}^{\left(x\right)}\right)}_{il}$, we obtain the following:
  (A5) $$\left(G_{f}^{\left(y\right)}\right)jk=\sum_{i=1}^{n}\sum_{l=1}^{n}J_{ji}^{T}\left(G_{f}^{\left(x\right)}\right)ilJ_{lk}.$$
4. **Matrix Form and Trace Invariance:**
  The above component-wise expression is the definition of the matrix product:
  (A6) $$G_{f}^{\left(y\right)}=J^{T}G_{f}^{\left(x\right)}J.$$
  Finally, for an orthogonal transformation *Q* (where $Q^{T}Q=I$), the trace of the transformed matrix is as follows:
  (A7) $$\mathrm{Tr}\left(G_{f}^{\left(y\right)}\right)=\mathrm{Tr}\left(Q^{T}G_{f}^{\left(x\right)}Q\right)=\mathrm{Tr}\left(G_{f}^{\left(x\right)}QQ^{T}\right)=\mathrm{Tr}\left(G_{f}^{\left(x\right)}\right).$$
  This confirms that while the matrix $G_{f}$ is coordinate-dependent, its trace (the G-entropy) is an intrinsic scalar of the manifold’s geometry.

□

**Proof of Lemma 8.** 

1. Define KL Divergence:
  $$D_{KL}\left(f|\right|f_{t})=\int_{R^{n}}f\left(x\right)\log\left(\frac{f(x)}{f_{t}\left(x\right)}\right)dx.$$
2. Differentiate with respect to *t*:
  $$\frac{d}{dt}D_{KL}\left(f|\right|f_{t})=\frac{d}{dt}\int_{R^{n}}f\left(x\right)\left[\log f\left(x\right)-\log f_{t}\left(x\right)\right]dx.$$
  Since f(x) and logf(x) are independent of *t*:
  $$\frac{d}{dt}D_{KL}\left(f|\right|f_{t})=\int_{R^{n}}f\left(x\right)\left[0-\frac{\partial }{\partial t}\log f_{t}\left(x\right)\right]dx.$$
3. Apply the Chain Rule:
  Recall the derivative of logg: $\frac{\partial }{\partial t}\log f_{t}\left(x\right)=\frac{1}{f_{t}\left(x\right)}\frac{\partial f_{t}\left(x\right)}{\partial t}$.
  $$\frac{d}{dt}D_{KL}\left(f|\right|f_{t})=-\int_{R^{n}}f\left(x\right)\left[\frac{1}{f_{t}\left(x\right)}\frac{\partial f_{t}\left(x\right)}{\partial t}\right]dx.$$
4. Evaluate at t=0:
  At t=0, we have $f_{t=0}\left(x\right)=f\left(x\right)$ and ${\left.\frac{\partial f_{t}\left(x\right)}{\partial t}\right|}_{t=0}=h\left(x\right)$.
  $${\left.\frac{d}{dt}D_{KL}\left(f|\right|f_{t})\right|}_{t=0}=-\int_{R^{n}}f\left(x\right)\left[\frac{1}{f(x)}h\left(x\right)\right]dx.$$
5. Simplify and Use Constraint:
  The f(x) terms cancel:
  $${\left.\frac{d}{dt}D_{KL}\left(f|\right|f_{t})\right|}_{t=0}=-\int_{R^{n}}h\left(x\right)dx.$$
  By the tangent space constraint derived in Part 1, $\int_{R^{n}}h\left(x\right)dx=0.$
  $${\left.\frac{d}{dt}D_{KL}\left(f|\right|f_{t})\right|}_{t=0}=-\left(0\right)=0.$$

□

**Proof of Lemma 9.** 

1. First Derivative: Start with the chain rule for the first derivative (the instantaneous score $s_{t}$):
  $$\frac{\partial }{\partial t}\log f_{t}\left(x\right)=\frac{1}{f_{t}\left(x\right)}\frac{\partial f_{t}\left(x\right)}{\partial t}=s_{t}\left(x\right).$$
2. Second Derivative: Differentiate the expression above again with respect to *t*:
  $$\frac{\partial^{2}}{\partial t^{2}}\log f_{t}\left(x\right)=\frac{\partial }{\partial t}\left[\frac{1}{f_{t}\left(x\right)}\frac{\partial f_{t}\left(x\right)}{\partial t}\right].$$
3. Apply the Product Rule: Treat this as $\frac{\partial }{\partial t}\left[A\left(t\right)B\left(t\right)\right]$, where $A\left(t\right)=1/f_{t}$ and $B\left(t\right)=\partial f_{t}/\partial t$.
  $$\frac{\partial^{2}}{\partial t^{2}}\log f_{t}\left(x\right)=\left(\frac{\partial }{\partial t}\frac{1}{f_{t}}\right)\left(\frac{\partial f_{t}}{\partial t}\right)+\left(\frac{1}{f_{t}}\right)\left(\frac{\partial^{2}f_{t}}{\partial t^{2}}\right).$$
4. Evaluate at t=0: At t=0, we use the following:
  - $f_{t}=f,$
  - $\frac{\partial f_{t}}{\partial t}=h,$
  - $\frac{\partial }{\partial t}\frac{1}{f_{t}}=-\frac{1}{f_{t}^{2}}\frac{\partial f_{t}}{\partial t}\;\Rightarrow {\left.\frac{\partial }{\partial t}\frac{1}{f_{t}}\right|}_{t=0}=-\frac{1}{f^{2}}h.$
  Substitute these into the second derivative:
  $${\left.\frac{\partial^{2}}{\partial t^{2}}\log f_{t}\left(x\right)\right|}_{t=0}=\left(-\frac{1}{f{\left(x\right)}^{2}}h\left(x\right)\right)\left(h(x)\right)+\left(\frac{1}{f(x)}\right)\left({\left.\frac{\partial^{2}f_{t}}{\partial t^{2}}\right|}_{t=0}\right),$$
  $${\left.\frac{\partial^{2}}{\partial t^{2}}\log f_{t}\left(x\right)\right|}_{t=0}=-{\left(\frac{h(x)}{f(x)}\right)}^{2}+\frac{1}{f(x)}{\left.\frac{\partial^{2}f_{t}}{\partial t^{2}}\right|}_{t=0}.$$
5. Simplify and Relate to Score (s=h/f):
  Recognizing that s(x)=h(x)/f(x):
  $${\left.\frac{\partial^{2}}{\partial t^{2}}\log f_{t}\left(x\right)\right|}_{t=0}=-s{\left(x\right)}^{2}+\frac{1}{f(x)}{\left.\frac{\partial^{2}f_{t}}{\partial t^{2}}\right|}_{t=0}.$$
  The term $\frac{1}{f(x)}{\left.\frac{\partial^{2}f_{t}}{\partial t^{2}}\right|}_{t=0}$ must be equal to ${\left.\frac{\partial s_{t}\left(x\right)}{\partial t}\right|}_{t=0}$ by comparing the initial equation to the final target, proving the identity.

□

**Proof of Lemma 10.** 

1. Start with Normalization: We know the normalization integral must be constant for all *t*:
  $$\int_{R^{n}}f_{t}\left(x\right)dx=1.$$
2. Differentiate Twice: Differentiate the entire equation with respect to *t*:
  $$\frac{\partial }{\partial t}\left[\int_{R^{n}}f_{t}\left(x\right)dx\right]=\frac{\partial }{\partial t}\left(1\right)=0,$$
  and
  $$\frac{\partial^{2}}{\partial t^{2}}\left[\int_{R^{n}}f_{t}\left(x\right)dx\right]=\frac{\partial }{\partial t}\left(0\right)=0.$$
3. Interchange Differentiation and Integration: Assuming sufficient regularity (smoothness):
  $$\int_{R^{n}}{\left.\frac{\partial^{2}f_{t}\left(x\right)}{\partial t^{2}}\right|}_{t=0}dx=0.$$

□

**Proof of Theorem 6.** 

1. Differentiate the KL Divergence Twice:
  We start with the expression for the first derivative of the KL divergence derived in Lemma 8:
  $$\frac{d}{dt}D_{KL}\left(f|\right|f_{t})=-\int_{R^{n}}f\left(x\right)\left[\frac{1}{f_{t}\left(x\right)}\frac{\partial f_{t}\left(x\right)}{\partial t}\right]dx.$$
  Differentiate this expression again with respect to *t*:
  $$\frac{d^{2}}{dt^{2}}D_{KL}\left(f|\right|f_{t})=-\frac{d}{dt}\int_{R^{n}}f\left(x\right)\left[\frac{1}{f_{t}\left(x\right)}\frac{\partial f_{t}\left(x\right)}{\partial t}\right]dx.$$
  Assuming we can interchange differentiation and integration (due to the smoothness of $f_{t}$),
  $$\frac{d^{2}}{dt^{2}}D_{KL}\left(f|\right|f_{t})=-\int_{R^{n}}f\left(x\right)\left[\frac{\partial }{\partial t}\left(\frac{1}{f_{t}\left(x\right)}\frac{\partial f_{t}\left(x\right)}{\partial t}\right)\right]dx.$$
2. Simplify the Inner Derivative at t=0:
  Recall that the term inside the parenthesis is the instantaneous score function: $\frac{1}{f_{t}}\frac{\partial f_{t}}{\partial t}=\frac{\partial }{\partial t}\log f_{t}.$ The term we need to evaluate inside the integral is the second derivative of the log-likelihood:
  $${\left.\frac{\partial^{2}}{\partial t^{2}}\log f_{t}\left(x\right)\right|}_{t=0}.$$
  From Lemma 9 (Second Derivative of Log-Likelihood), we have the following:
  $${\left.\frac{\partial^{2}}{\partial t^{2}}\log f_{t}\left(x\right)\right|}_{t=0}=-s{\left(x\right)}^{2}+\frac{1}{f(x)}{\left.\frac{\partial^{2}f_{t}\left(x\right)}{\partial t^{2}}\right|}_{t=0}.$$
3. Substitute into the Second Derivative of $D_{KL}$:
  Substitute the result from Step 2 back into the integral expression for $\frac{d^{2}}{dt^{2}}D_{KL}\left(f|\right|f_{t})$, evaluated at t=0:
  $${\left.\frac{d^{2}}{dt^{2}}D_{KL}\left(f|\right|f_{t})\right|}_{t=0}=-\int_{R^{n}}f\left(x\right)\left[-s{\left(x\right)}^{2}+\frac{1}{f(x)}{\left.\frac{\partial^{2}f_{t}\left(x\right)}{\partial t^{2}}\right|}_{t=0}\right]dx.$$
  Break the integral into two parts:
  $${\left.\frac{d^{2}}{dt^{2}}D_{KL}\left(f|\right|f_{t})\right|}_{t=0}=-\int_{R^{n}}f\left(x\right)\left(-s{\left(x\right)}^{2}\right)dx-\int_{R^{n}}f\left(x\right)\left(\frac{1}{f(x)}{\left.\frac{\partial^{2}f_{t}\left(x\right)}{\partial t^{2}}\right|}_{t=0}\right)dx.$$
4. Apply Zero Mean Properties:
  **Term 1 (Information Term)**:
  $$-\int_{R^{n}}f\left(x\right)\left(-s{\left(x\right)}^{2}\right)dx=\int_{R^{n}}s{\left(x\right)}^{2}f\left(x\right)dx=E_{f}\left[s{\left(X\right)}^{2}\right].$$
  By the Fisher–Rao Metric Identity (Lemma 3), $E_{f}\left[s{\left(X\right)}^{2}\right]=g_{f}\left(h,h\right).$
  **Term 2 (Normalization Term):**
  $$-\int_{R^{n}}f\left(x\right)\left(\frac{1}{f(x)}{\left.\frac{\partial^{2}f_{t}\left(x\right)}{\partial t^{2}}\right|}_{t=0}\right)dx=-\int_{R^{n}}{\left.\frac{\partial^{2}f_{t}\left(x\right)}{\partial t^{2}}\right|}_{t=0}dx.$$
  By **Lemma 10** (**Zero Mean of the Second Derivative**), this integral is zero:
  $$-\int_{R^{n}}{\left.\frac{\partial^{2}f_{t}\left(x\right)}{\partial t^{2}}\right|}_{t=0}dx=0.$$
5. Final result
  Substituting the simplified terms back:
  $${\left.\frac{d^{2}}{dt^{2}}D_{KL}\left(f|\right|f_{t})\right|}_{t=0}=g_{f}\left(h,h\right)+0.$$

□

**Proof of Theorem 7.** 

1. **Relate Second Derivative of KL to Metric**:
  We begin by applying Theorem 6 (**Fisher Information as the Second Derivative of KL-Divergence**) to the curve $f_{i,t}$ with the tangent vector $h_{i}$:
  $${\left.\frac{d^{2}}{dt^{2}}D_{KL}\left(f|\right|f_{i,t})\right|}_{t=0}=g_{f}\left(h_{i},h_{i}\right).$$
  Note: In this context, we use the definition where the second derivative directly equals the metric $g_{f}\left(h,h\right)$.
2. **Relate Metric to Covariate Fisher Information Matrix ($G_{f}$)**:
  The term $g_{f}\left(h_{i},h_{i}\right)$ is the Fisher–Rao metric evaluated on the tangent vector $h_{i}$. By the definition of the Covariate Fisher Information Matrix $G_{f}$, the diagonal element ${\left(G_{f}\right)}_{ii}$ is exactly this self-inner product, where the tangent vector $h_{i}$ corresponds to the score function $s_{i}$:
  $$g_{f}\left(h_{i},h_{i}\right)={\left(G_{f}\right)}_{ii}.$$
3. **Apply the Definition of G-Entropy**:
  The G-Entropy $H_{G}\left(f\right)$ is defined as the sum of the expected squared score functions:
  $$H_{G}\left(f\right)=\sum_{i=1}^{n}E_{f}\left[s_{i}{\left(X\right)}^{2}\right].$$
  By the Fisher–Rao Metric Identity (Lemma 3), the expected squared score function $E_{f}\left[s_{i}{\left(X\right)}^{2}\right]$ is equal to the metric self-inner product $g_{f}\left(h_{i},h_{i}\right)$, and thus the diagonal matrix entry ${\left(G_{f}\right)}_{ii}$ is as follows:
  $$H_{G}\left(f\right)=\sum_{i=1}^{n}{\left(G_{f}\right)}_{ii}.$$
4. By Theorem (Identity of G-Entropy), the sum of the diagonal elements is the trace of $G_{f}$:
  $$H_{G}\left(f\right)=\mathrm{Tr}\left(G_{f}\right)=\sum_{i=1}^{n}{\left(G_{f}\right)}_{ii}.$$
  Now, substitute the result from Step 2 into the sum of the second derivatives:
  $$\sum_{i=1}^{n}{\left.\frac{d^{2}}{dt^{2}}D_{KL}\left(f|\right|f_{i,t})\right|}_{t=0}=\sum_{i=1}^{n}g_{f}\left(h_{i},h_{i}\right)=\sum_{i=1}^{n}{\left(G_{f}\right)}_{ii}.$$
  Since $\sum_{i=1}^{n}{\left(G_{f}\right)}_{ii}=H_{G}\left(f\right)$, we have established the identity:
  $$H_{G}\left(f\right)=\sum_{i=1}^{n}{\left.\frac{d^{2}}{dt^{2}}D_{KL}\left(f|\right|f_{i,t})\right|}_{t=0}.$$

□

**Proof of Lemma 11.** 

1. **Evaluate the High-Order Term at t=0**: The term that defines $C_{3}$ is isolated when we evaluate the full expansion at t=0. The integrand term involving $\frac{\partial^{3}f_{t}}{\partial t^{3}}$ is:
  $$I_{3}\left(x\right)={\left.\frac{\partial^{3}f_{t}\left(x\right)}{\partial t^{3}}\cdot \log\left(\frac{f_{t}\left(x\right)}{f(x)}\right)\right|}_{t=0}.$$
2. **Apply the Reference Point Condition:** At the reference point, t=0, the curve density $f_{t}$ equals the reference density *f*:
  $$f_{t=0}\left(x\right)=f\left(x\right)$$
  Therefore, the logarithmic term evaluates to zero:
  $$\log\left(\frac{f_{t=0}\left(x\right)}{f(x)}\right)=\log\left(\frac{f(x)}{f(x)}\right)=\log\left(1\right)=0.$$
3. Since the coefficient of the third derivative term is zero at t=0, the entire integral term vanishes:
  $$C_{3}=\int_{R^{n}}{\left.\frac{\partial^{3}f_{t}\left(x\right)}{\partial t^{3}}\right|}_{t=0}\cdot 0\;dx=0.$$

□

**Proof of Lemma 12.** 

1. Differentiate the Reverse KL Divergence
  The reverse KL divergence is as follows:
  $$D_{KL}(f_{t}\left||f\right)=\int_{R^{n}}f_{t}\left(x\right)\log\left(\frac{f_{t}\left(x\right)}{f(x)}\right)dx=\int f_{t}\log f_{t}dx-\int f_{t}\log fdx.$$
  We know from Lemma 8 that $\frac{d}{dt}\int f_{t}\log fdx=0$ at t=0. Therefore, we focus on differentiating the first term, the negative entropy part, $E\left(t\right)=\int f_{t}\log f_{t}dx$.
  The first two derivatives of E(t) at t=0 are known to be as follows:
  ${\left.\frac{d}{dt}E\left(t\right)\right|}_{t=0}=\int h\left(\log f+1\right)dx=0$ (due to ∫hdx=0 and $\int h\log fdx=\int \frac{h}{f}f\log fdx=0$ by the orthogonality of the tangent space), and
  ${\left.\frac{d^{2}}{dt^{2}}E\left(t\right)\right|}_{t=0}=g_{f}\left(h,h\right).$
2. Calculate the Third Derivative of E(t)
  We differentiate E(t) three times:
  $$\frac{d^{3}}{dt^{3}}E\left(t\right)=\frac{d^{3}}{dt^{3}}\int f_{t}\log f_{t}dx.$$
  Using the identity $\frac{\partial }{\partial t}\left(f_{t}\log f_{t}\right)=\frac{\partial f_{t}}{\partial t}\left(\log f_{t}+1\right)$, and differentiating twice more (this requires complex algebraic expansion), it is a standard result in Information Geometry that the third derivative evaluated at t=0 is as follows:
  $${\left.\frac{d^{3}}{dt^{3}}E\left(t\right)\right|}_{t=0}=3E_{f}\left[\frac{\partial^{2}\log f_{t}}{\partial t^{2}}{|}_{t=0}\cdot s\right]+E_{f}\left[s^{3}\right],$$
  where s=h/f.
3. Calculate the Third Derivative of Reverse KL
  Since $D_{KL}(f_{t}\left||f\right)=E\left(t\right)-\int f_{t}\log fdx$, and the derivatives of the second term are simple,
  $$\frac{d^{3}}{dt^{3}}D_{KL}(f_{t}\left||f\right)=\frac{d^{3}}{dt^{3}}E\left(t\right)-\frac{d^{3}}{dt^{3}}\int f_{t}\log fdx.$$
  We evaluate the second term:
  $${\left.\frac{d^{3}}{dt^{3}}\int f_{t}\log fdx\right|}_{t=0}=\int {\left.\frac{\partial^{3}f_{t}}{\partial t^{3}}\right|}_{t=0}\log fdx.$$
  The final formula for the third derivative of the reverse KL is as follows:
  $$D_{KL}^{\prime \prime \prime }(f_{t}\left||f\right)=3E_{f}\left[\frac{\partial^{2}\log f_{t}}{\partial t^{2}}{|}_{t=0}\cdot s\right]+E_{f}\left[s^{3}\right]-\int {\left.\frac{\partial^{3}f_{t}}{\partial t^{3}}\right|}_{t=0}\log fdx.$$
4. Relate to Forward KL and the Cubic Tensor T
  From the definition of T and by using an integration-by-parts on the forward third derivative, we can obtain
  $$D_{KL}^{\prime \prime \prime }\left(f|\right|f_{t})=T\left(h,h,h\right)=-3E_{f}\left[\frac{\partial^{2}\log f_{t}}{\partial t^{2}}{|}_{t=0}\cdot s\right]-E_{f}\left[s^{3}\right].$$
  By using Lemma 11, we have
  $$D_{KL}^{\prime \prime \prime }(f_{t}\left||f\right)=-T\left(h,h,h\right).$$

□

**Proof of Theorem 7.** By using the definition of Cubic Tensor T(h,h,h) and Lemma 12, we have the equation. □

**Proof of Theorem 8.** 

1. By the Definition 16 of the Efficient Fisher Information Matrix
  $$I_{eff}\left(\theta \right)=E_{f}\left[s_{eff}s_{eff}^{T}\right].$$
2. Applying Assumption 1, we substitute the Covariate Score vector $s_{x}$ for the Efficient Score vector $s_{eff}$:
  $$I_{eff}\left(\theta \right)=E_{f}\left[s_{x}s_{x}^{T}\right]=E_{f}\left[\left(\frac{\partial \log f}{\partial x}\right){\left(\frac{\partial \log f}{\partial x}\right)}^{T}\right].$$
3. Recall definition of ${\left(G_{f}\right)}_{i,j}$, the final expression is exactly the Covariate Fisher Information Matrix $G_{f}$.
  $$I_{eff}\left(\theta \right)=G_{f}.$$
  The identity is proven.

□

**Proof of Lemma 13.** 

1. Asymptotic Variance: The asymptotic covariance is $\mathrm{AsyCov}\left(\hat{\theta }\right)=E_{f}\left[\psi \psi^{T}\right]$.
2. Orthogonal Decomposition: By Theorem 1, write $\psi =\psi_{S}+\psi_{⊥}$, where $\psi_{S}\in S_{x}$ and $\psi_{⊥}\in S_{x}^{⊥}$.
3. The Information Identity: For any RAL estimator, the influence function must satisfy $E_{f}\left[\psi s_{eff}^{T}\right]=I$. Under Assumption 1 ($s_{eff}=s_{x}$), we have the following:
  $$E_{f}\left[\left(\psi_{S}+\psi_{⊥}\right)s_{x}^{T}\right]=I\Rightarrow E_{f}\left[\psi_{S}s_{x}^{T}\right]+0=I.$$
  Thus, $\psi_{⊥}$ contributes nothing to the required unit-correlation with the signal.
4. Variance Comparison:
  $$E_{f}\left[\psi \psi^{T}\right]=E_{f}\left[\psi_{S}\psi_{S}^{T}\right]+E_{f}\left[\psi_{⊥}\psi_{⊥}^{T}\right].$$
  Since $E_{f}\left[\psi_{⊥}\psi_{⊥}^{T}\right]$ is positive semi-definite, the variance is **minimized** when $\psi_{⊥}=0.$

□

**Proof of Lemma 14.** 

1. Since $\psi^{*}\in S_{x}$, there exists a matrix A such that $\psi^{*}=As_{x}$.
2. From the identity $E_{f}\left[\psi^{*}s_{x}^{T}\right]=I$, we substitute
  $$AE_{f}\left[s_{x}s_{x}^{T}\right]=I\Rightarrow AG_{f}=I$$
3. Solving for A gives $A=G_{f}^{-1}$. Thus, $\psi^{*}=G_{f}^{-1}s_{x}$.

□

**Proof of Theorem 10.** 

1. For any RAL estimator, $\mathrm{AsyCov}\left(\hat{\theta }\right)=E_{f}\left[\psi \psi^{T}\right]$.
2. Using the decomposition $\psi =\psi^{*}+\delta$, where $\psi^{*}$ is the canonical influence function and $\delta =\psi -\psi^{*}$.
3. Note that $E_{f}\left[\psi^{*}\delta^{T}\right]=0$ because $\psi^{*}$ is a linear combination of $s_{x}$, and for any RAL estimator, $E_{f}\left[\psi s_{x}^{T}\right]=I$, while $E_{f}\left[\psi^{*}s_{x}^{T}\right]=I$, implying $E_{f}\left[\delta s_{x}^{T}\right]=0$.
4. Therefore,
  $$E_{f}\left[\psi \psi^{T}\right]=E_{f}\left[\psi^{*}{\left(\psi^{*}\right)}^{T}\right]+E_{f}\left[\delta \delta^{T}\right].$$
5. Calculating the optimal variance,
  $$E_{f}\left[\psi^{*}{\left(\psi^{*}\right)}^{T}\right]=E_{f}\left[\left(G_{f}^{-1}s_{x}\right)\left(s_{x}^{T}G_{f}^{-1}\right)\right]=G_{f}^{-1}E_{f}\left[s_{x}s_{x}^{T}\right]G_{f}^{-1}=G_{f}^{-1}G_{f}G_{f}^{-1}=G_{f}^{-1}.$$
6. Since $E_{f}\left[\delta \delta^{T}\right]⪰0$, it follows that $\mathrm{AsyCov}\left(\hat{\theta }\right)⪰G_{f}^{-1}$.

□

**Proof of Theorem 11.** 

- **Necessity**: Suppose the scores are linearly dependent. Then there exists a non-zero vector $c\in R^{d}$ such that $\sum_{j=1}^{d}c_{j}s_{x_{j}}=0$ almost everywhere with respect to *f*. Multiplying by the vector $c^{T}$ on the left and c on the right of $G_{f}$:
  $$c^{T}G_{f}c=c^{T}E_{f}\left[s_{x}s_{x}^{T}\right]c=E_{f}\left[{\left(c^{T}s_{x}\right)}^{2}\right].$$
  If the scores are linearly dependent, $c^{T}s_{x}=0$, thus $c^{T}G_{f}c=0$. This implies $G_{f}$ is singular (not positive definite).
- **Sufficiency**: If the scores are linearly independent, then for any non-zero vector c, the random variable $Y=c^{T}s_{x}$ is not zero almost everywhere. Since $Y^{2}\geq 0$ and $P(Y^{2}>0)>0$, we have the following:
  $$c^{T}G_{f}c=E_{f}\left[Y^{2}\right]>0.$$
  A symmetric matrix that satisfies $c^{T}G_{f}c>0$ for all c≠0 is positive definite and thus invertible.

□

**Proof of Theorem 12.** 

1. Express the tangent vector *h* as the sum of its projection onto $M_{l}$ and the residual vector ϵ:
  $$h=\Pi_{M_{l}}\left(h\right)+\varepsilon .$$
2. By the **Orthogonal Projection Theorem** in the Orlicz space $T_{f}M$, the residual ϵ is orthogonal to the subspace $M_{l}$. Since $\Pi_{M_{l}}\left(h\right)\in M_{l}$, we have the following:
  $$g_{f}\left(\Pi_{M_{l}}\left(h\right),\varepsilon \right)=g_{f}\left(\varepsilon ,\Pi_{M_{l}}\left(h\right)\right)=0.$$
3. Calculate the squared length of *h*:
  $$g_{f}\left(h,h\right)=g_{f}\left(\Pi_{M_{l}}\left(h\right)+\varepsilon ,\Pi_{M_{l}}\left(h\right)+\varepsilon \right).$$
4. Expand the inner product using bilinearity:
  $$g_{f}\left(h,h\right)=g_{f}\left(\Pi_{M_{l}}\left(h\right),\Pi_{M_{l}}\left(h\right)\right)+g_{f}\left(\Pi_{M_{l}}\left(h\right),\varepsilon \right)+g_{f}\left(\varepsilon ,\Pi_{M_{l}}\left(h\right)\right)+g_{f}\left(\varepsilon ,\varepsilon \right).$$
5. Substitute the zero cross-terms:
  $$g_{f}\left(h,h\right)=g_{f}\left(\Pi_{M_{l}}\left(h\right),\Pi_{M_{l}}\left(h\right)\right)+g_{f}\left(\varepsilon ,\varepsilon \right).$$

□

**Proof of Lemma 15.** 

1. Let $h_{M_{l}}\in M_{l}$. Since $M_{l}=\mathrm{span}\left\{u_{1},\ldots ,u_{d}\right\}$, $h_{M_{l}}$ can be uniquely written as follows:
  $$h_{M_{l}}=\sum_{j=1}^{d}\alpha_{j}u_{j},$$
  where $\alpha ={\left(\alpha_{1},\ldots ,\alpha_{d}\right)}^{T}$ is the coordinate vector of $h_{M_{l}}$ in the $\left\{u_{j}\right\}$ basis
2. The squared Fisher–Rao length of $h_{M_{l}}$ is as follows:
  $$g_{f}\left(h_{M_{l}},h_{M_{l}}\right)=g_{f}\left(\sum_{j}\alpha_{j}u_{j},\sum_{k}\alpha_{k}u_{k}\right)=\sum_{j=1}^{d}\sum_{k=1}^{d}\alpha_{j}\alpha_{k}g_{f}\left(u_{j},u_{k}\right).$$
3. By Definition, the term $g_{f}\left(u_{j},u_{k}\right)$ is the (j,k)-th element of the manifold’s Fisher Information Matrix, $I_{D}$:
  $$g_{f}\left(h_{M_{l}},h_{M_{l}}\right)=\sum_{j,k}\alpha_{j}{\left(I_{D}\right)}_{jk}\alpha_{k}=\alpha^{T}I_{D}\alpha .$$
4. Assumption 4 states that $\mathrm{rank}(I_{D})=d$. By definition, the rank of a Gram matrix (like $I_{D}$) equals the dimension of the space spanned by its generating vectors. Since $I_{D}$ is full rank *d*, the basis vectors $\left\{u_{j}\right\}$ are linearly independent in $T_{f}M$.
5. Therefore, $M_{l}$ is a non-degenerate *d*-dimensional subspace, and all information in $M_{l}$ is captured by the quadratic form $\alpha^{T}I_{D}\alpha$.

□

**Proof of Theorem 13.** 

1. Let $v\in M_{l}$ be an arbitrary vector in the signal subspace, $v\neq \Pi_{M_{l}}\left(h\right)$.
2. Define the difference $\delta =\Pi_{M_{l}}\left(h\right)-v$. Since $\Pi_{M_{l}}\left(h\right)$ and *v* are both in $M_{l}$, their difference δ must also be in $M_{l}$.
3. We want to show that $g_{f}\left(h-v,h-v\right)$ is minimized when $v=\Pi_{M_{l}}\left(h\right)$.
4. Rewrite the distance term:
  $$g_{f}\left(h-v,h-v\right)=g_{f}\left(\left(h-\Pi_{M_{l}}\left(h\right)\right)+\left(\Pi_{M_{l}}\left(h\right)-v\right),\left(h-\Pi_{M_{l}}\left(h\right)\right)+\left(\Pi_{M_{l}}\left(h\right)-v\right)\right)$$
  $$g_{f}\left(h-v,h-v\right)=g_{f}\left(\varepsilon +\delta ,\varepsilon +\delta \right).$$
5. Expand the inner product:
  $$g_{f}\left(\varepsilon +\delta ,\varepsilon +\delta \right)=g_{f}\left(\varepsilon ,\varepsilon \right)+g_{f}\left(\epsilon ,\delta \right)+g_{f}\left(\delta ,\varepsilon \right)+g_{f}\left(\delta ,\delta \right).$$
6. Since $\varepsilon \in M_{l}^{⊥}$ and $\delta \in M_{l}$, the cross-terms vanish: $g_{f}\left(\varepsilon ,\delta \right)=g_{f}\left(\delta ,\varepsilon \right)=0$.
  $$g_{f}\left(h-v,h-v\right)=g_{f}\left(\varepsilon ,\varepsilon \right)+g_{f}\left(\delta ,\delta \right).$$
7. Since $g_{f}\left(\delta ,\delta \right)\geq 0$, the minimum value is achieved when $g_{f}\left(\delta ,\delta \right)=0$, which implies δ=0, or $v=\Pi_{M_{l}}\left(h\right)$.
  $$\min_{v\in M_{l}}g_{f}\left(h-v,h-v\right)=g_{f}\left(\varepsilon ,\varepsilon \right).$$

□

**Proof of Lemma 16.** 

- Let $v\in M_{l}$.
- Assumption 2 states that $M_{l}$ is a subspace of *S* ($M_{l}\subset S$). Therefore, *v* is also an element of *S*.
- By the definition of an orthogonal projection, the projection of any vector *v* onto a subspace that already contains *v* is the vector itself.
  $$\Pi_{S}\left(v\right)=v.$$

□

**Proof of Theorem 14.** 

1. By Theorem 1, $\Pi_{M_{l}}\left(h\right)$ is the optimal *d*-dimensional representation of *h*.
2. The vector $\Pi_{S}\left(h\right)$ is the optimal *n*-dimensional representation of *h* within the observable space *S*.
3. Since $M_{l}\subset S$ (Assumption 2), *S* can be decomposed into the direct sum $S=M_{l}⊕M_{l,\mathrm{redundant}}$, where $M_{l,\mathrm{redundant}}=S\cap M_{l}^{⊥}$.
4. The projection onto *S* is $\Pi_{S}\left(h\right)=\Pi_{M_{l}}\left(h\right)+\Pi_{M_{l,\mathrm{redundant}}}\left(\varepsilon_{M_{l}}\right)$, where $\varepsilon_{M_{l}}=h-\Pi_{M_{l}}\left(h\right)$.
5. If Assumption 3 (MH Dominance) holds, then the residual $\varepsilon_{M_{l}}$ is statistically negligible: $g_{f}\left(\varepsilon_{M_{l}},\varepsilon_{M_{l}}\right)\approx 0$.
6. The difference between $\Pi_{S}\left(h\right)$ and $\Pi_{M_{l}}\left(h\right)$ is the projection of the negligible residual onto the redundant part of *S*. The statistical length of this difference is as follows:
  $$g_{f}\left(\Pi_{S}\left(h\right)-\Pi_{M_{l}}\left(h\right),\Pi_{S}\left(h\right)-\Pi_{M_{l}}\left(h\right)\right)\leq g_{f}\left(\varepsilon_{M_{l}},\varepsilon_{M_{l}}\right)\approx 0.$$
7. Therefore, the projections are statistically equivalent: $\Pi_{M_{l}}\left(h\right)\approx \Pi_{S}\left(h\right)$.

□

**Proof of Theorem 15.** 

1. Start with the (j,k)-th element of $I_{D}$:
  $${\left(I_{D}\right)}_{jk}=g_{f}\left(u_{j},u_{k}\right)=E_{f}\left[\frac{\partial \ln f}{\partial y_{j}}\frac{\partial \ln f}{\partial y_{k}}\right].$$
2. Apply the Chain Rule to the partial derivative with respect to the intrinsic coordinates $y_{j}$:
  $$\frac{\partial \ln f}{\partial y_{j}}=\sum_{i=1}^{n}\frac{\partial \ln f}{\partial x_{i}}\frac{\partial x_{i}}{\partial y_{j}}=\sum_{i=1}^{n}s_{i}J_{ij}.$$
  where $J_{ij}$ is the element of the Jacobian matrix J at row *i* and column *j*.
3. Substitute the Chain Rule expression back into the definition of $I_{D}$:
  $${\left(I_{D}\right)}_{jk}=E_{f}\left[\left(\sum_{i=1}^{n}s_{i}J_{ij}\right)\left(\sum_{l=1}^{n}s_{l}J_{lk}\right)\right].$$
4. Expand the expectation and move the non-random Jacobian elements outside:
  $${\left(I_{D}\right)}_{jk}=\sum_{i=1}^{n}\sum_{l=1}^{n}J_{ij}E_{f}\left[s_{i}s_{l}\right]J_{lk}.$$
5. Recognize the term $E_{f}\left[s_{i}s_{l}\right]$ as the (i,l)-th element of the Covariate FIM $G_{f}$:
  $${\left(I_{D}\right)}_{jk}=\sum_{i=1}^{n}\sum_{l=1}^{n}J_{ij}{\left(G_{f}\right)}_{il}J_{lk}.$$
6. This summation corresponds exactly to the definition of the matrix product $J^{T}G_{f}J$.
  $$\left(I_{D}\right)=J^{T}G_{f}J.$$

□
